# The Impact of Asking Intention or Self-Prediction Questions on Subsequent Behavior: A Meta-Analysis

**DOI:** 10.1177/1088868315592334

**Published:** 2015-07-10

**Authors:** Chantelle Wood, Mark Conner, Eleanor Miles, Tracy Sandberg, Natalie Taylor, Gaston Godin, Paschal Sheeran

**Affiliations:** 1 University of Sheffield, UK; 2 University of Leeds, UK; 3 University of Sussex, UK; 4 Macquarie University, Sydney, Australia; 5 Laval University, Quebec City, Quebec, Canada; 6 University of North Carolina at Chapel Hill, USA

**Keywords:** question–behavior effect, mere-measurement effect, self-prophecy effect, meta-analysis, behavior change

## Abstract

The current meta-analysis estimated the magnitude of the impact of asking intention and self-prediction questions on rates of subsequent behavior, and examined mediators and moderators of this question–behavior effect (QBE). Random-effects meta-analysis on 116 published tests of the effect indicated that intention/prediction questions have a small positive effect on behavior (*d*_+_ = 0.24). Little support was observed for attitude accessibility, cognitive dissonance, behavioral simulation, or processing fluency explanations of the QBE. Multivariate analyses indicated significant effects of social desirability of behavior/behavior domain (larger effects for more desirable and less risky behaviors), difficulty of behavior (larger effects for easy-to-perform behaviors), and sample type (larger effects among student samples). Although this review controls for co-occurrence of moderators in multivariate analyses, future primary research should systematically vary moderators in fully factorial designs. Further primary research is also needed to unravel the mechanisms underlying different variants of the QBE.

The question–behavior effect (QBE) refers to the impact of asking questions about a behavior (vs. not asking such questions) on subsequent performance of that behavior. This effect is also known as the mere-measurement effect, the self-prophecy effect, self-erasing errors of prediction, and self-generated validity (Sprott, Spangenberg, Block, et al., 2006). An illustrative study by Williams, Block, and Fitzsimons (2006) showed that asking students about their intentions to exercise increased subsequent self-reported exercise rates from 14% to 26% 2 months later. Although the QBE has also been assessed using questions about attitudes and past behavior, the vast majority of studies ask self-prediction or intention questions, which form the focus of the present review. We report a meta-analysis that updates and extends previous narrative (Dholakia, 2010) and quantitative (Spangenberg & Greenwald, 1999; Sprott, Spangenberg, Knuff, & Devezer, 2006) reviews, and tests conceptual and methodological moderators using both univariate and multivariate analyses.

The QBE has been observed in a variety of research domains, and most often for health, consumer, and prosocial behaviors. Within the health domain, a large number of studies have demonstrated that the QBE can be harnessed as an effective intervention, increasing uptake of health checks (Conner, Godin, Norman, & Sheeran, 2011; Spangenberg & Sprott, 2006; Sprott, Smith, Spangenberg, & Freson, 2004; Sprott, Spangenberg, & Fisher, 2003), health screening (Sandberg & Conner, 2009), and vaccinations (Conner et al., 2011). QBEs on consumer purchase behaviors have also been demonstrated and replicated (Chandon, Morwitz, & Reinartz, 2004; Fitzsimons & Williams, 2000; Janiszewski & Chandon, 2007; Morwitz & Fitzsimons, 2004; Morwitz, Johnson, & Schmittlein, 1993; Van Kerckhove, Geuens & Vermeir, 2012), as have effects on prosocial behaviors such as blood donation (Cioffi & Garner, 1998; Godin, Sheeran, Conner, & Germain, 2008).

The most recent meta-analyses of the QBE are either long out of date (Spangenberg & Greenwald, 1999) or focused only on health-related behaviors (Sprott, Spangenberg, Knuff, & Devezer, 2006). Importantly, neither meta-analysis specifically examined mediators and moderators of the QBE, which form the focus of the present review. Sprott, Spangenberg, Block, et al. (2006), and Dholakia (2010) pointed out that there have been few attempts to examine the moderators and drivers of the QBE *across* different domains of research. Similarly, although a number of plausible theoretical explanations for the QBE have been supported in the literature, very few studies have pitted these explanations against one another, and there is no current consensus as to the mechanisms underlying the QBE. Accordingly, the time seems fitting for a broad meta-analytic synthesis that includes recent literature, with the aim of shedding light on mediators and moderators of the QBE.

## Mechanisms Underlying the QBE

A number of mediators of the QBE have been proposed as potential mechanisms. The most prominent explanations involve attitude accessibility (Morwitz et al., 1993; Morwitz & Fitzsimons, 2004), cognitive dissonance (Spangenberg & Greenwald, 1999; Spangenberg & Sprott, 2006; Spangenberg, Sprott, Grohmann, & Smith, 2003), and processes related to behavioral simulation and processing fluency (Janiszewski & Chandon, 2007; Levav & Fitzsimons, 2006; Sherman, 1980). We discuss these key explanations of the QBE, and how they are tested in the current meta-analysis, below.

### Attitude Accessibility

The attitude accessibility explanation of the QBE assumes that asking people to report their behavioral intentions or to predict their behavior activates the attitude underlying that behavior, making it more accessible in memory. In turn, this heightened accessibility of the relevant attitude increases the likelihood that the person will perform the target behavior (Dholakia, 2010) or, more accurately, makes it more likely that the person will act consistently with his or her attitude (Morwitz et al., 1993; Morwitz & Fitzsimons, 2004). It is well established that the relationship between attitudes and behavior is stronger when attitudes are more accessible (Chen & Bargh, 1999; Fazio, Chen, McDonel, & Sherman, 1982; Fazio & Williams, 1986; for a meta-analysis, see Cooke & Sheeran, 2004). Attitude accessibility accounts of the QBE suggest that questioning should only promote behavior when the sample predominantly holds attitudes that favor performance of the behavior (Fitzsimons & Morwitz, 1996; Godin et al., 2008; Morwitz et al., 1993). This explanation of the QBE is particularly prevalent in research using the mere-measurement label (Dholakia, 2010).

Consistent with an attitude accessibility explanation of the QBE, participants who are asked to report their intentions or to predict their behavior exhibit more accessible attitudes relative to those that are not asked (Chapman, 2001; Fitzsimons, Nunes, & Williams, 2007; Morwitz & Fitzsimons, 2004). Wood, Conner, Sandberg, Godin, and Sheeran (2014) also recently observed that attitude accessibility was a significant mediator of the relationship between intention measurement and behavior. Other research has also shown that the valence of attitudes toward the behavior moderate the QBE in line with an attitude accessibility account, such that participants reporting positive attitudes show a stronger QBE than those with negative attitudes (Conner et al., 2011). Indeed, some studies show that questions can decrease performance of the behavior among participants with negative attitudes (e.g., Conner et al., 2011, Study 2).

However, support for attitude accessibility as a mediator of the QBE is by no means uniform. For example, both Perkins, Smith, Sprott, Spangenberg, and Knuff, (2008), and Spangenberg et al. (2012) found no significant differences in attitude accessibility between participants asked to predict whether they would engage in a target behavior relative to participants who made no such prediction. Furthermore, some demonstrations of the QBE occur under conditions that are not easily accounted for by attitude accessibility. In particular, attitude accessibility does not provide a wholly satisfying explanation of the QBE for behaviors that are performed a long time after questioning, when transient increases in attitude accessibility presumably have decayed. For instance, Godin et al. (2008) observed QBE effects up to 12 months after questioning.

In the current meta-analysis, we test the attitude accessibility explanation by assessing the influence of an index of attitude accessibility on the strength of the QBE. Too few studies reported response latency measures of attitude accessibility to permit a quantitative synthesis, so it was necessary to measure accessibility indirectly. The attitude accessibility index used here had two components—the valence of the attitude and the proportion of participants whose attitude was activated. The attitude valence component was measured by independent ratings of attitude for the focal behavior and sample. The attitude activation component was based on the assumption that attitudes are more activated when participants answer the relevant prediction/intention questions. In field studies of the QBE, questionnaires are distributed and returned (or not) and subsequent behavior for the entire sample is measured, irrespective of whether or not participants responded to the questionnaire (e.g., Godin et al., 2010; Godin et al., 2008). However, as not all participants answer the relevant prediction/intention questions (i.e., complete and return the questionnaire), the response rate to the questionnaire provides an index of the proportion of participants whoseattitudes are activated. For each study, therefore, the attitude accessibility index was computed by multiplying the response rate to the questionnaire by the attitude valence rating. The association between attitude accessibility and the magnitude of the QBE was then assessed via meta-regression.

### Cognitive Dissonance

Cognitive dissonance (Festinger, 1957) is the dominant explanation of the QBE among researchers using the self-prophecy label (Dholakia, 2010). Festinger (1957) defined cognitive dissonance as “the existence of nonfitting relations among cognitions” where cognitions include “any knowledge, opinion or belief about the environment, about oneself, or about one’s behavior” (p. 3). Cognitive dissonance is a tension state that motivates attempts to reduce dissonance. In the context of the QBE, cognitive dissonance accrues when people perform a behavior that is inconsistent with a relevant standard of judgment, that is, when people’s actions are inconsistent with beliefs about how they should act (Stone & Cooper, 2001). Answering prediction/intention questions increases the salience of both social norms associated with the behavior (a standard of judgment) and any previous failures to behave in a manner that is consistent with such norms. Such questions cause the respondent to become simultaneously cognizant of what they should do and what they have done in the past; any observed inconsistency can potentially generate cognitive dissonance. One way to reduce this aversive cognitive dissonance is to subsequently act in accordance with the social norms or standards (Aronson, 1992). In their self-standards model of cognitive dissonance, Stone and Cooper (2001) noted that both normative standards (i.e., perceived norms) and personal standards (i.e., individual attitudes) can operate in similar ways as the standard for judgment. Dholakia (2010) suggested that personal goals or resolutions can also serve as standards.

Consistent with the dissonance account of the QBE, Spangenberg et al. (2012) found that participants who were asked to predict their own behavior were more likely to engage in downward comparisons that are known to reduce dissonance (Spangenberg et al., 2003)—Participants subsequently reported higher levels of past socially desirable behavior for themselves, but lower levels for other people. However, most research concerning the cognitive dissonance explanation of the QBE has evaluated this mechanism only indirectly. Support for the mechanisms involved in the QBE is often inferred from moderator effects that are more consistent with one theoretical approach rather than another (Sprott, Spangenberg, Block, et al., 2006), and not by directly measuring the experience of dissonance. These indirect tests offer mixed support for the role of cognitive dissonance (Spangenberg & Sprott, 2006; Spangenberg et al., 2003; Spangenberg et al., 2012). Spangenberg et al. (2003) found that a self-affirmation manipulation (that is known to reduce cognitive dissonance) attenuated the QBE, whereas Sprott et al. (2003) found that preference for consistency (which is known to increase susceptibility to cognitive dissonance; Cialdini, Trost, & Newsom, 1995) did not moderate the QBE.

The current meta-analysis sought to address the paucity of direct evidence concerning the cognitive dissonance mechanism. We therefore rated the likely degree of discomfort (i.e., cognitive dissonance) experienced by participants at the time of prediction, if their past behavior was not consistent with the normative or personal standards conveyed by their self-predictions or intentions concerning their future behavior. Greater experienced dissonance should increase the likelihood of acting in line with normative/personal standards. This explanation of the QBE assumes that actions consistent with standards will reduce dissonance, whereas actions that are inconsistent with standards could maintain or even increase dissonance. Accordingly, we also rated the likely degree of discomfort participants would experience if their future behavior was not consistent with their predictions/intentions—both at the time of prediction and at the moment of enacting the behavior. Given the potential for overlap among these three ratings, we first examined their intercorrelation before determining whether to treat the items individually or as a combined index of cognitive dissonance.

### Behavioral Simulation and Processing Fluency

A third explanation of the QBE concerns the processes involved in the simulation of behavior and related effects on processing fluency. In the original demonstration of this effect, Sherman (1980) suggested that the QBE is driven by the formation of cognitive representations or behavioral scripts during questioning that are subsequently reactivated when the individual has an opportunity to enact the behavior. Sherman proposed that mental simulation may lead to an increase in the accessibility of the behavioral script or in the perceived likelihood of behavior; either process would promote behavior consistent with the representation.

Evidence that ease of representation influences the QBE provides indirect support for the role of behavioral simulation. Similar to Sherman (1980), Levav and Fitzsimons (2006) argued that being asked to predict future behavior leads participants to mentally represent that behavior. However, Levav and Fitzsimons suggest that participants subsequently consider how easy the behavior was to represent; greater ease of representation is misinterpreted as an increased likelihood of the behavior’s occurrence, which is subsequently translated into an increase in actual behavior. Levav and Fitzsimons’s ease of representation hypothesis therefore suggests that the QBE should be attenuated for behaviors that are more difficult to represent or simulate. Consistent with this idea, Levav and Fitzsimons found that manipulating ease of representation had systematic effects on the QBE. For example, the use of question frames that are congruent with the likely attitude toward behavior (i.e., asking participants with likely negative attitudes toward fatty food about their intentions to *avoid eating* fatty foods) may be easier to represent than incongruent question frames (i.e., asking the same participants about their intentions *to eat* fatty foods), and congruent framing generated larger QBEs (Levav & Fitzsimons, 2006). In this meta-analysis, we evaluate the role of ease of representation by examining whether the congruence between the question frame (approach, avoidance, or both approach and avoidance) and likely attitude distribution moderates the QBE. Evidence for the moderating effect of ease of representation would provide indirect support for a behavioral simulation explanation for the QBE.

Recent work has provided more direct support for the role of processing fluency in enhancing the accessibility or perceived likelihood of behavior. Janiszewski and Chandon (2007) argued that the QBE prompts processing fluency effects in the form of transfer-appropriate processing. At the moment of acting, activation of the behavior representation and processes involved in deciding whether to act should be facilitated because the same behavior representation and processes are accessed when participants predicted behavior. Janiszewski and Chandon suggest that this increased processing fluency may be misinterpreted as an increased probability of the behavior actually occurring, that is, as an inclination toward the behavior that serves to change subsequent behavior. Consistent with this explanation, Janiszewski and Chandon found larger QBEs when there was greater correspondence between the intention and behavior measures. That is, asking participants to predict general or specific consumer behaviors influenced general and specific ratings of likelihood to purchase, respectively. However, general predictions did not increase the likelihood of specific purchases, or vice versa. In the current meta-analysis, we use the principle of correspondence (e.g., Fishbein & Ajzen, 1975) to quantify the match between questions and behavior (along the dimensions of target, action, context, and time), to examine the effect of processing fluency on the QBE. It is important to note, however, that the role of correspondence is not unique to processing fluency accounts for the QBE. Sprott et al. (2004) argued that if cognitive dissonance underlies the QBE, then people who make predictions about a specific behavior should be more likely to capitalize on the first opportunity to reduce dissonance by changing that particular behavior. Thus, both processing fluency and cognitive dissonance accounts predict a larger QBE for intention/prediction questions that show greater correspondence with the behavioral measure. Among QBE studies that use similar self-report measures to tap intention/prediction and behavior, greater correspondence may also reflect greater common method variance (see Conner, Warren, Close, & Sparks, 1999, for an example in relation to intention and behavior). To examine whether any overall effect of correspondence observed in the full set of studies reviewed is due to the common method variance of using self-reports, we also test effects of correspondence in the subset of studies that used objective measures of behavior.

## Characteristics of the Question, Behavior, and Methodology as Moderators of the QBE

In addition to exploring conceptual factors specified by key explanations of the QBE, we also examined features of the primary studies to identify potential moderators of the QBE—to shed light on the QBE’s boundary conditions and to increase understanding of how to magnify (or attenuate) the effect. In particular, the current meta-analysis explored the impact of characteristics of the question, behavior, and methodology on the strength of the QBE.

### Question Characteristics

#### Question type

QBE research under the mere-measurement versus self-prophecy label differs in the type of question posed to respondents (Sprott, Spangenberg, Block, et al., 2006; Williams et al., 2006). Mere-measurement studies generally ask participants to report their *intentions* to engage in behavior, whereas self-prophecy studies characteristically ask participants to *predict*, or rate the likelihood of performing, the behavior. There are reasons to suspect that prediction questions could have a larger effect on subsequent behavior than intention questions. Self-predictions are probability judgments about what one will do and are based on appraisals of both the feasibility *and* desirability of acting. Behavioral intentions, however, are based more on the behavior’s desirability than its feasibility (Sheppard, Hartwick, & Warshaw, 1988) and represent an idealized aim to reach a goal that may involve little commitment. Evidence indicates that behavioral intentions relevant to people’s overarching identity goals are enacted with little tenacity or effort (Gollwitzer, Sheeran, Michalski, & Seifert, 2009), and primary research and meta-analysis have shown that self-prediction has a larger correlation with subsequent behavior compared with intention (Armitage & Conner, 2001; Armitage, Norman, Alganem, & Conner, 2015; Sheppard et al., 1988). Here, we test whether question type (prediction vs. intention) moderates the QBE.

#### Questions related to the theory of planned behavior (TPB) and anticipated regret

Different questions have been used to manipulate the QBE in different behavioral domains. In studies of health and prosocial behaviors, participants are typically asked not only about their intentions but also about their attitudes, subjective norms, and perceived behavioral control in relation to the focal behavior—the cognitions held to determine intentions by the TPB (e.g., Godin, Bélanger-Gravel, Amireault, Vohl, & Perusse, 2011; Godin et al., 2008; Sandberg & Conner, 2009). Activating behavioral, normative, and control considerations alongside reporting one’s intentions could lead to an increased QBE. Alternatively, if only intention questions drive the QBE, the additional items in TPB questions could be distracting and attenuate the QBE.

Several studies have examined the impact of measuring anticipated regret alongside intentions (e.g., Sandberg & Conner, 2009, 2011), based on findings that measuring anticipated regret strengthens intentions and improves intention–behavior consistency (Abraham & Sheeran, 2003, 2004). The QBE might thus be increased by including questions about anticipated regret. However, it is also possible that drawing attention to anticipated regret could be perceived as blatant manipulation by participants and lead to reactance (Brehm, 1966) that reduces the QBE (Godin et al., 2010; see also Godin, Germain, Conner, Delage, & Sheeran, 2014). The current meta-analysis examines whether the QBE is moderated by the use of questions based on the TPB, or the addition of questions relating to anticipated regret.

#### Number of questions

Studies vary in terms of the number of intention or prediction questions to which participants are asked to respond. However, there has been little explicit consideration of whether the number of prediction or intention items moderates the magnitude of the QBE (though see Chapman, 2001; Morwitz et al., 1993), and whether the total number of general questions relating to the focal behavior is influential. The hypothesis that the number of intention/prediction questions strengthens the QBE would seem to be consistent with attitude accessibility and behavioral simulation explanations of the QBE, as repeated measurement should make attitudes and behavioral scripts more accessible or fluent. The current meta-analysis therefore tests whether the number of self-prediction or behavioral intention items, and the total number of behavior-related items moderate the QBE.

### Behavior Characteristics

#### Experience with the behavior

The QBE has been observed for both novel (e.g., singing the Star Spangled Banner over the telephone; Sherman, 1980) and familiar behaviors (e.g., flossing teeth, eating fatty foods; Levav & Fitzsimons, 2006; Williams, Fitzsimons, & Block, 2004). However, relatively little research has directly examined whether experience with performing a behavior moderates the QBE, and the evidence is mixed. Morwitz et al. (1993) found that the magnitude of the QBE on PC purchase was stronger for participants with no previous experience with owning a PC, whereas Godin et al. (2010; Godin et al., 2008) found that the QBE was stronger for participants with considerable experience of blood donation than for participants with little or no previous experience. This meta-analysis will test the role of experience in predicting the strength of the QBE.

#### Behavior domain

The question of whether the QBE constitutes a behavior change intervention with wide reach demands analysis of the nature of the target behavior. There has been a recent debate concerning the operation of the QBE in relation to risky behaviors (see Fitzsimons & Moore, 2008b; Sherman, 2008, for detailed discussions). Some studies show that the QBE decreases performance of risky behaviors (e.g., consumption of fatty foods; Levav & Fitzsimons, 2006), whereas other studies show increases in risky behaviors (e.g., illegal drug use; Williams et al., 2006). Such doubt over the direction of the QBE for risky behaviors has led some researchers to recommend that asking questions about these behaviors should be avoided—so that risk incidence is not exacerbated (e.g., Fitzsimons & Moore, 2008a). Gollwitzer and Oettingen (2008) argued, however, that a meta-analytic comparison of the QBE for risky, as compared with other, behaviors is required before conclusions are reached. Accordingly, the current meta-analysis tests the magnitude of the QBE as a function of the risk level of the target behavior, by examining the impact of behavior domain on the QBE.

#### Social desirability

QBE studies have investigated both socially desirable (e.g., blood donation) and socially undesirable (e.g., eating fatty foods) behaviors. Evidence concerning the influence of social desirability on the QBE is mixed. Whereas some studies suggest that the direction of the QBE depends on the desirability of the target behavior (Williams et al., 2004), other research suggests that the QBE prompts increases in behavior regardless of social desirability (Fitzsimons et al., 2007; Williams et al., 2006). In the current meta-analysis, we attempt to resolve this debate by testing whether the rated social desirability of each behavior moderates the QBE.

#### Difficulty of behavior

Another issue concerns whether the magnitude of the QBE depends on the difficulty of performing the target behavior. Classic theories of motivation (e.g., Atkinson, 1954) point out that behavioral performance depends not only on the desirability of the perceived outcomes but also on the feasibility of performing the behavior. This might suggest either a linear effect (i.e., QBE is smaller for difficult-to-perform behaviors relative to those that are easier to perform) or a curvilinear effect (i.e., moderately difficult behaviors are most motivating and therefore likely to produce a larger QBE relative to behaviors that are easy to perform). We therefore assess both linear and quadratic relationships between behavioral difficulty and effect sizes.

#### Behavior frequency

It is also unclear whether the magnitude of the QBE is equivalent for repeatedly performed behaviors (e.g., frequency of flossing; Williams et al., 2004; frequency of gym attendance; Spangenberg, 1997) and one-off behaviors (e.g., questionnaire return; Chapman, 2001; flu vaccination uptake; Conner et al., 2011). Ouellette and Wood (1998) suggested that infrequent behaviors are guided more by conscious intentions than are frequent behaviors, where performance may be habitual and automatic. Thus, a larger QBE might be expected for infrequent or rare behaviors relative to more frequently performed behaviors.

#### Behavior measure

The QBE has been observed both using self-report (e.g., participants indicate whether or how often they performed the target behavior, via questionnaire: Fitzsimons et al., 2007; Williams et al., 2006; Williams et al., 2004) and objective measures (e.g., observation of actual behavior; Chandon et al., 2004; Conner et al., 2011; Williams et al., 2004) of behavior. A number of studies have measured the same behavior using both objective and self-report indices, allowing for a direct test of whether the size of the QBE is affected by the way in which behavior is assessed. However, findings are inconsistent. Whereas some research suggests that the QBE is not affected by the type of behavioral measure (Williams et al., 2004), other studies show stronger QBEs using objective measures (Sprott, Spangenberg, & Perkins, 1999), and still other studies observe stronger effects using self-report measures of behavior (Spence, Burgess, Rodgers, & Murray, 2009). The present meta-analysis assesses the impact of behavior measure (self-report vs. objective) on the QBE.

### Methodological Characteristics

#### Time interval

Another important factor related to deployment of the QBE as a behavior change intervention concerns the durability of behavioral effects in the wake of self-prediction or intention measurement. Research has observed a significant QBE when behavior is measured shortly after questioning within a single laboratory session (e.g., Janiszewski & Chandon, 2007; Morwitz & Fitzsimons, 2004), over delays of 1 or 2 weeks (e.g., Cioffi & Garner, 1998; Williams et al., 2004), and also after more extended time periods of up to 12 months (e.g., Godin et al., 2008). However, only a handful of studies compared the same behavior at different time points within a single study, and these studies obtained inconsistent results (see, for example, Godin et al., 2008; Spangenberg, 1997). The current meta-analysis examines whether the time interval between questioning and behavior influences the magnitude of the QBE.

#### Personal contact with the experimenter

Garnering self-predictions or intentions from participants can involve personal contact with the experimenter (e.g., questions answered via telephone or face-to-face), or no personal contact (e.g., questions answered via written or online surveys). Concerns about making a good impression on the researcher could steer predictions and intentions in a socially desirable direction when they are reported via personal contact. Moreover, reporting intentions and predictions in-person to the experimenter could increase people’s feelings of commitment or obligation, and motivate greater striving for consistency between their intentions/predictions and their future behavior. We therefore test whether the size of the QBE differs in studies that involve personal contact during questioning relative to studies with no personal contact.

#### Research setting

The QBE may differ for studies conducted in laboratory versus field settings. In particular, the QBE could be larger in laboratory settings due to greater demand effects: Participants could feel pressure to act consistently with their intentions or self-predictions because of the presence of the experimenter or the authority of the setting. We therefore compare the magnitude of the QBE as a function of the research setting.

#### Response rate and incentives

One might also expect a smaller effect in field-based relative to laboratory studies because of lower survey response rates in field settings. Research within the health domain, for example, suggests that significant QBEs are found only for those participants who complete and return questionnaires (i.e., when per-protocol analysis is used to examine the data, Sandberg & Conner, 2009). Accordingly, the response rate to questionnaires in field studies (the proportion of participants returning the questionnaire) will be investigated as a potential moderator in the present meta-analysis. We also examine whether the use of incentives (participant payments) moderates the size of the QBE. For example, use of incentives may increase the response rate to questionnaires in field studies (which, as above, is expected to increase the size of the QBE).

#### Sample type

We examine whether recruitment of students versus non-students as participants influences the QBE. Reliance on student samples has been criticized in psychological research (Henrich, Heine, & Norenzayan, 2010). However, the QBE has been tested in a wide variety of different populations, and there seems no reason to expect differences in the QBE as a function of this sample characteristic. Nonetheless, we also code and test the type of sample as a moderator.

#### Publication year

Finally, we investigate whether there is any evidence of a “decline effect” in the QBE literature (see, for example, Schooler, 2011), by examining whether publication year influences the magnitude of the QBE.

## The Present Research

Although a great deal of research has been conducted on the QBE, there is little consensus concerning the mechanisms that underlie the effect. The potential for harnessing the QBE as a simple but effective behavior change intervention is constrained by our relative lack of knowledge regarding the factors that moderate the effect. The present research aims to tackle this gap in the literature by deploying meta-analytic techniques both to gauge the overall effect of questioning on behavior and evaluate theoretical and methodological factors that may determine the magnitude and direction of the QBE.

## Method

### Literature Search

A search of published research articles was undertaken using ISI Web of Science (encompassing Science Citation Index Expanded, Social Science Citation Index Expanded, Arts and Humanities Citation Index) and PsychInfo, encompassing the period between the first published article on the QBE (Sherman, 1980) and March 5, 2013. Search terms used were *self-generated validity, measurement reactivity, self-erasing errors of prediction, mere-measurement effect, self-prophecy effect, question–behavior effect*, and *questionnaire effects on behavior*.^1^ The reference lists of each article and key narrative reviews published in the area (i.e., Dholakia, 2010) were examined, and contact was also made with leading authors in the field. The review was restricted to published studies to ensure that we could retrieve the necessary information concerning mediators and moderators of the QBE from the primary reports. It is well established that published reports exhibit higher methodological quality than unpublished reports (e.g., Egger, Jüni, Bartlett, Holenstein, & Sterne, 2003); unpublished work may be partial or incomplete, and it may not be feasible to retrieve the necessary information from authors of unpublished reports. This inclusion criterion seemed justified as our primary aim was to identify mediators and moderators of the QBE, and not merely estimate the overall effect size.

**Figure 1.**
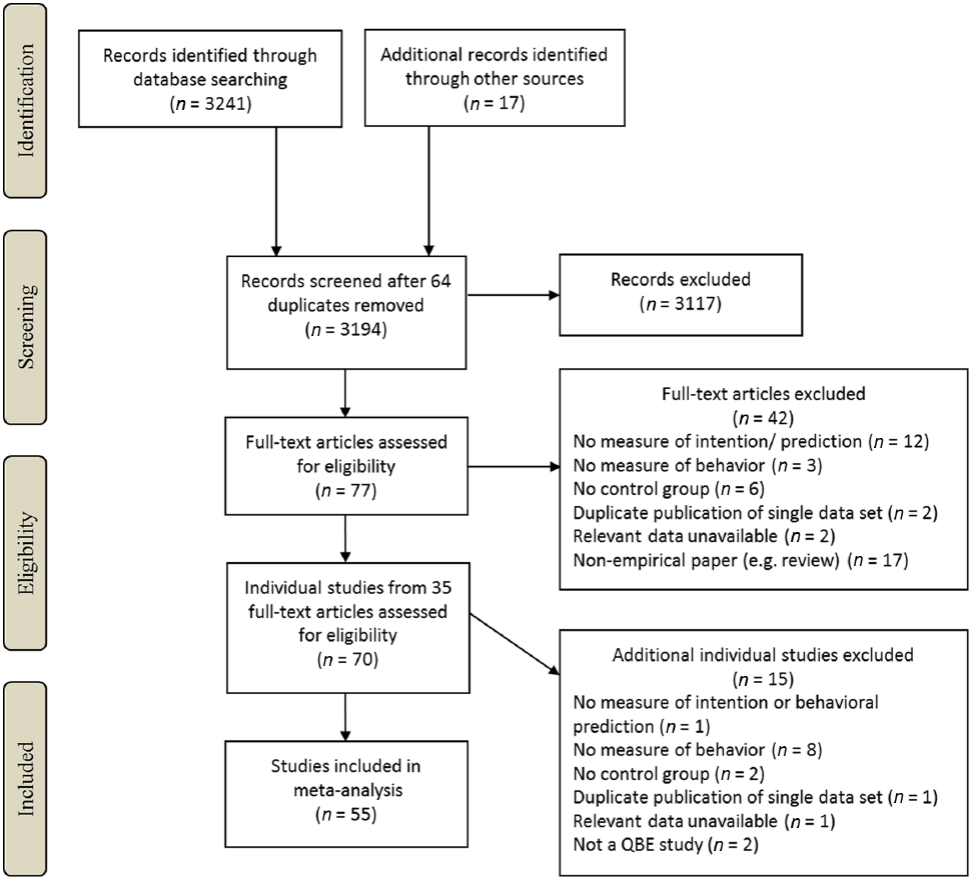
Literature search flow diagram. *Note.* QBE = question–behavior effect.

Figure 1 shows the flow of articles through the literature search. After screening for eligibility as detailed below, 35 articles consisting of a total of 55 study data sets were suitable for inclusion, comprising 116 tests of the QBE. Included articles are marked with an asterisk in the “Reference” section of this article.

### Inclusion Criteria and Eligibility

Studies had to meet the following inclusion criteria: First, studies had to address the QBE in their research, as indicated by their use of relevant QBE terminology (i.e., use of the terms *question–behavior effect, mere measurement, self-generated validity, self-prophecy effect*, or *self-erasing nature of errors of prediction*). Second, to allow the QBE to be quantified, studies had to include a measure of behavior. Third, studies had to include a condition that measured participant’s behavioral intentions, their expectations about the likelihood of them performing a target behavior, or predictions of their future behavior, as well as an appropriate comparison control condition (i.e., no intentions/expectations measured, or non-target behavior intentions measured). Finally, studies had to provide sufficient statistical information to permit calculation of effect sizes. In the case of missing or incomplete effect size data, authors were contacted to request the relevant statistics.

There were also a number of exclusion criteria. QBE studies that did not directly invite a response to questions (e.g., using hypothetical “ask yourself” questions; Fitzsimons & Shiv, 2001; Spangenberg et al., 2003) were excluded from the data set, as were those that focused on measurement of other cognitions that did not require prediction of future behavior, such as expectations regarding satisfaction with or evaluation of customer service (e.g., Dholakia & Morwitz, 2002; Dholakia, Singh, & Westbrook, 2010; Ofir & Simonson, 2007) and measurement of past behavior (e.g., Falk, 2010). Manipulations that involved predicting the behavior of another person (and not the self) were also excluded (Levav & Fitzsimons, 2006, Experiment 1). Finally, to avoid contamination effects, any manipulations or conditions that confounded the QBE with other interventions designed to change behavior, such as commitment to a self-reward (Fitzsimons et al., 2007), motivational interventions (Ayres et al., 2013), or implementation intentions (Fitzsimons et al., 2007; Godin et al., 2010) were also excluded.^2^

#### Non-independent observations

Several studies reported more than one behavioral observation for the same group of participants (e.g., behavior measured at two time points, different ways of measuring a single behavior, multiple alternative methods of analysis). However, inclusion of non-independent observations risks underestimating the error variance associated with each effect size (Borenstein, Hedges, Higgins, & Rothstein, 2009). In cases where the inclusion of multiple non-independent measures did not enhance the scope of the main analyses or was irrelevant for moderator analyses, we elected to select a single key observation for inclusion and omit any additional observations. The key observation selected for inclusion was either that identified by the authors of each study as their key measure or, in the absence of this information, the first measure reported. Similarly, in cases where multiple control groups were compared with a single experimental group and inclusion of more than one control condition provided no added information concerning the QBE, the “purest” control group only was included (i.e., the no-contact control groups; Cioffi & Garner, 1998, Experiment 1; Obermiller, Spangenberg, & Atwood, 1992, Experiment 1; Sherman, 1980, Experiment 1; the neutral questionnaire control group; Chapman, 2001; the no-question control group; Van Kerckhove et al., 2012, Experiment 2a).

A number of studies also reported results using multiple methods of analysis or data screening. Sandberg and Conner (2009, Experiment 1), Godin et al. (2010, Experiment 1), and Conner et al. (2011, Experiment 1 and Experiment 2), for example, reported the results of both per-protocol analyses (i.e., including data from participants who completed the questionnaire only) and intention-to-treat analyses (i.e., including data from all participants irrespective of whether or not they undertook the intervention and completed the questionnaire). In a similar vein, Greenwald, Carnot, Beach, and Young (1987, Experiment 1 and Experiment 2); Williams et al. (2006, Experiment 1); and Spangenberg (1997, Experiment 1) reported analyses using different screening criteria. Here, the most conservative analysis that excluded the fewest participants (e.g., intention-to-treat analysis) was selected for inclusion.

In some cases, however, non-independent data were integral to the moderator analyses as when behavior was measured over several different time periods within the same sample (Chandon et al., 2004, Experiment 1; Chapman, 2001, Experiment 2; Godin et al., 2010, Experiment 1; Godin et al., 2008, Experiment 1; Spangenberg, 1997, Experiment 1), when objective and self-report measures were both used (Spence et al., 2009, Experiment 1), or when studies compared multiple independent experimental groups with a single control group (Cioffi & Garner, 1998; Lawrence & Ferguson, 2012, Experiment 3; Sprott et al., 2004, Experiment 2, Williams et al., 2004, Experiment 4). Calculating an average effect size that collapses over the observations (see Higgins & Green, 2011, Section 16.5.4) is not appropriate in these cases, as so doing would result in the omission of important moderator data. Accordingly, effect sizes for each of these non-independent comparisons were included to preserve moderator data. However, to avoid underestimating the error variance associated with each effect size, the sample sizes used to calculate the standard errors for each group were divided by the number of times they were included (see Higgins & Green, 2011, Section 16.5.4; Michie, Abraham, Whittington, McAteer, & Gupta, 2009; Webb, Miles, & Sheeran, 2012).^3^ The observations included in the meta-analysis are presented in Appendices A and B in the article’s online Supplemental Materials.

### Data Extraction and Calculation of Effect sizes

The effect size used was the standardized mean difference Cohen’s *d*, with a Hedge’s *g* correction (Borenstein et al., 2009). Effect sizes were calculated using the formulas described in Borenstein et al. (2009); *d* was calculated by dividing the difference between means by the pooled standard deviation, and the *g* adjustment was calculated using the formula, *d*(1 − [3] / [4*df* − 1]). Although we use the term *d* to describe this effect, it is also referred to as *g* (Borenstein et al., 2009), or *d*_unbiased_ or *d*_unb_ (Fritz, Morris, & Richler, 2012).

For continuous data, effect sizes were calculated using the means, standard deviations, and sample sizes for each group whenever possible. If these data were missing or incomplete, authors were contacted for further information and/or *d* was alternatively calculated using *t* values, univariate *F* values, and existing reported effect size measures (e.g., *r*). For dichotomous data, *d* was first calculated using the number or rate of events and sample size for each group. When these data were unavailable, effect size was instead calculated using odds ratios and confidence limits or chi-square and total sample size. If sample sizes for each condition needed to calculate effect sizes were not reported or not provided by the authors, the number of participants in each condition was estimated by dividing the total sample size by the number of conditions. Effect sizes were calculated such that a positive effect size represents an increase in behavior following positively framed questions (e.g., “I intend to give blood during the next 6 months”; Godin et al., 2008) or neutral questions framed in terms of dichotomous or contrasting options (e.g., “How likely or unlikely would you be to try a Canadian candy bar if it was available in the U.S.”; Morwitz & Fitzsimons, 2004), or a decrease in behavior following negatively framed questions (e.g., the likelihood of not eating fatty foods; Levav & Fitzsimons, 2006), relative to the control condition (i.e., no intentions/predictions measured, or intentions/predictions measured for a non-target behavior). Cohen’s (1992) guidelines were used to interpret effect sizes, such that effect sizes of .30, .50, and .80 represented small, medium, and large effects, respectively.

### Moderator Coding

Each moderator variable was independently coded by one of the first five authors or the seventh author. Moderators that could be directly extracted from the data were recorded by the first author. These moderators are as follows: Whether questions based on the TPB or anticipated regret were included, the number of behavioral intention or prediction questions, the total number of behavioral questions, behavior frequency, the type of behavior measure, time interval, research setting, response rate and use of incentives, sample type, and publication year. For the remaining moderators, 20% of the studies were double-coded by another author. Interrater reliability was measured using Cohen’s kappa for categorical variables, and intraclass correlations (ICCs) for continuous variables, and is reported below. Disagreements were resolved by discussion (see Appendices A and B in the article’s online Supplemental Materials for key theoretical and methodological moderator coding and effect sizes of studies included in the meta-analysis).

#### Mechanisms underlying the QBE

Studies were coded for response rate and attitude valence (to compute the index of attitude accessibility), cognitive dissonance, ease of representation, and correspondence. Interrater reliability was acceptable for all indices and is reported below. Attitude valence was indexed by rating how positive most of the sample’s attitude toward the behavior was likely to be (5-point scale; *extremely negative* to *extremely positive*; ICC = .62). The attitude accessibility index was then calculated by standardizing the attitude valence rating and the response rate to the question, which was the proportion of participants who had returned the relevant questionnaire containing the QBE manipulation (laboratory studies had a response rate of 1.0, whereas field studies varied), and multiplying these to form a single interaction term, such that higher scores indicate greater accessibility of more positive attitudes. Cognitive dissonance was indexed by three items. The first item rated the degree of discomfort experienced by the sample at the moment of prediction, if their past behavior is inconsistent with their predictions or intentions regarding future behavior (5-point scale; *not at all uncomfortable* to *very uncomfortable*; ICC = .66). The second and third items rated the degree of discomfort experienced by participants at the moment of prediction and the moment of behavior, respectively, if their future behavior were to be inconsistent with their predictions or intentions regarding that behavior (5-point scale; *not at all uncomfortable* to *very uncomfortable*; ICCs = .70 and .78). Cronbach’s alpha indicated a high degree of consistency across the three items (α = .89). Accordingly, the three items were averaged to form a single index of cognitive dissonance.

Following Levav and Fitzsimons (2006), ease of representation was operationalized in terms of the congruency between the sample’s likely attitude and framing of the question in approach, avoidance, or both approach and avoidance, terms (5-point scale; *very incongruent match* to *very congruent match*; ICC = .94). For example, approach-framed questions asking about a behavior likely characterized by a strong positive attitude would score 5, whereas avoidance-framed questions asking about a behavior likely characterized by a strong positive attitude would score 1. Questions framed in terms of both approach and avoidance (e.g., Do you predict that (a) You will do X or (b) You will not do X?) scored 3. Finally, correspondence between the measure of intention or behavioral prediction and behavior was indexed by assigning one point for each match in terms of action, target, context, and time (ICC = .89;for example, Fishbein & Ajzen, 1975).

#### Question characteristics

Question type was coded *behavioral intention, prediction*, or *mixed* (κ = 1.0). Questions were classified as assessing intention items if they asked participants to report their intentions or plans, for example, “Do you intend to donate during the Summer Drive?” (Cioffi & Garner, 1998). Questions were instead classified as requesting behavioral prediction if they asked participants to predict their behavior (e.g., “Overall, do you predict that in the next 6 days: (a) You will [exercise, or watch the news, or read books] or (b) You will not [exercise, or watch the news, or read books]”; Chandon, Smith, Morwitz, Spangenberg, & Sprott, 2011), to estimate the likelihood of performing a particular behavior (e.g., “How likely or unlikely would you be to try a New Zealand candy bar if it was available in the U.S.?” Fitzsimons & Williams, 2000), or used other terminology implying prediction or expectation (e.g., “Would you return to the clinic 1 month after the first shot for a free second hepatitis vaccine shot?” Cox, Cox, Cyrier, Graham-Dotson, & Zimet, 2012). Finally, studies were classified as mixed if they included both intention and prediction items, or items that were not clearly classifiable. Where no exact wording was reported, the item was coded in line with the terminology used by the authors. We also recorded whether studies included TPB questions (attitudes, subjective norms, or perceived behavioral control), questions concerning anticipated regret, the number of items used to report behavioral intentions or self-predictions, and the total number of questions that participants were asked about the behavior.

#### Behavior characteristics

Behaviors were coded according to the following characteristics: experience with the behavior, behavior domain, social desirability, difficulty of behavior, behavior frequency, and the type of behavior measure. Experience with behavior was classified into one of three categories according to the sample’s likely level of experience of performing the behavior (*none has experience, experience varies in the sample, or all highly experienced*; κ = 0.83). The behavior domain was classified as *health, prosocial, consumer, undesirable or risky behavior, and other* (κ = 1.0). Behaviors were classified as healthy if performing them was conceived to promote the health or well-being of the individual performing the behavior (e.g., health club attendance and exercise; Chandon et al., 2011; Godin et al., 2011). Behaviors were coded as prosocial if performing them was considered to benefit other people or society as a whole (e.g., behaviors related to donating blood; Cioffi & Garner, 1998; Godin et al., 2010; Godin et al., 2008). Consumer behaviors were classified as such if they involved consumer purchase or choice decisions (e.g., purchase from an online grocery company; Chandon et al., 2004). Behaviors were classified as risky or undesirable if performing them was considered to be detrimental to health or well-being, or otherwise socially undesirable (e.g., illegal drug use; Williams et al., 2006). Behaviors that did not fit clearly into the above categories were classified as “other” (e.g., reading; Chandon et al., 2011; Levav & Fitzsimons, 2006).

Social desirability was indexed by rating how much the sample would think that other people would want them to perform the behavior (5-point scale; other people would very much [*not want–want*] the participants to perform this behavior; ICC = .89). The difficulty of performing the behavior was indexed by rating how hard it would be for the sample to perform the behavior (5-point scale; *not at all hard* to *very hard*; ICC = .61). Behavior frequency (*one-time vs. repeated*) and the type of behavior measure (*self-report* [e.g., questionnaire] *vs. objective* [e.g., medical records, behavioral observation]) was also coded.

#### Methodological characteristics

The following data were also extracted from each study: time interval, personal contact with the experimenter, research setting, response rate and use of incentive, sample type, and publication year. Time interval was indexed by the number of days between questioning and behavioral measurement. Contact with the experimenter was classified as personal when intentions or predictions were requested by the experimenter either face-to-face or via telephone, or impersonal when requested via paper questionnaires, online questionnaires, or lab-based computer tasks (κ = 1.0). The research setting was either the laboratory or the field. The response rate to questioning was the proportion of participants who had returned the relevant questionnaire containing the QBE manipulation; laboratory studies therefore always had a response rate of 1, whereas the values in field studies varied. Whether or not participants received a payment for taking part in the study was also recorded. Finally, sample type was coded *student, non-student*, or *mixed*.

### Analysis Strategy

Comprehensive Meta-Analysis software, Version 2.2.064 (Borenstein, Hedges, Higgins, & Rothstein, 2005) was used to compute effect sizes and conduct all univariate analyses. Weighted average effect sizes (*d*_+_) were based on a random-effects model, following general recommendations for the use of random-effects analyses in meta-analyses (Field, 2003), and given study differences are unlikely to be fully explained by a small number of simple study characteristics (Cooper & Hedges, 1994). Cochran’s (1952) *Q* statistic was used to test heterogeneity of effect sizes, with a significant *Q* indicative of significant heterogeneity. The *I*^2^ statistic (Higgins & Thompson, 2002) was used to quantify the degree of heterogeneity. *I*^2^ reflects the percentage variance in effect sizes that cannot be explained by chance, and *I*^2^ values of 25%, 50%, and 75% indicate low, moderate, and high levels of heterogeneity, respectively (Higgins, Thompson, Deeks, & Altman, 2003).

Moderator analyses of categorical variables were undertaken using random-effects subgroup analyses. Variance between studies was expected to be consistent across subgroups, and thus, the heterogeneity variance within each subgroup (τ^2^) was estimated by a single value collapsing across subgroups (Borenstein et al., 2009). In any one moderator analysis, subgroups with fewer than five observations were excluded from analysis. Moderator analyses of continuous variables were undertaken using random-effects method of moments meta-regression. The statistical significance of each moderator was assessed using *Q* tests analogous to ANOVA, such that a significant between-groups *Q* indicates that the effect size differs significantly as a function of the moderator (Borenstein et al., 2009). The proportion of heterogeneity accounted for by each moderator was computed using adjusted *R*^2^, which represents the ratio of variance explained by the moderator relative to the amount of variance in total, calculated using the following formula: (1 − [τ^2^ within / τ^2^ total]) (Borenstein et al., 2009).

Multivariate analyses were performed in STATA (release 12; Stata Corporation, College Station, TX, the United States), using the same statistical model as Comprehensive Meta-Analysis Version 2.2.064 (method of moments without Knapp–Hartung modification of standard errors). Continuous moderators were standardized before analysis to reduce multicollinearity.

Publication bias was evaluated via inspection of funnel plots, Egger’s regression test (Egger, Smith, Schneider, & Minder, 1997), and Duval and Tweedie’s (2000) Trim and Fill technique, which computes an adjusted effect size based on the inclusion of putative missing studies.

## Results

### Overall Effect Size

The meta-analysis was conducted on 116 tests of the QBE (*N* = 54,985, unadjusted sample sizes). Meta-analysis using a random-effects model demonstrated a corrected mean-weighted effect size of 0.24, with confidence intervals (CIs) not including 0 (95% CI = [0.18, 0.30]). Interpreted in the context of Cohen’s (1992) guidelines, this suggests that intention/prediction questions have a small, reliable, and positive effect on subsequent behavior. The *Q* statistics revealed evidence of significant heterogeneity in effect sizes (*Q* = 379.55, *p* < .001). The *I*^2^ value was 69.70%, which constitutes a moderate-to-high degree of heterogeneity according to Higgins et al.’s (2003) guidelines. The *Q* and *I*^2^ results thus indicate that moderator analysis is justified.

**Figure 2.**
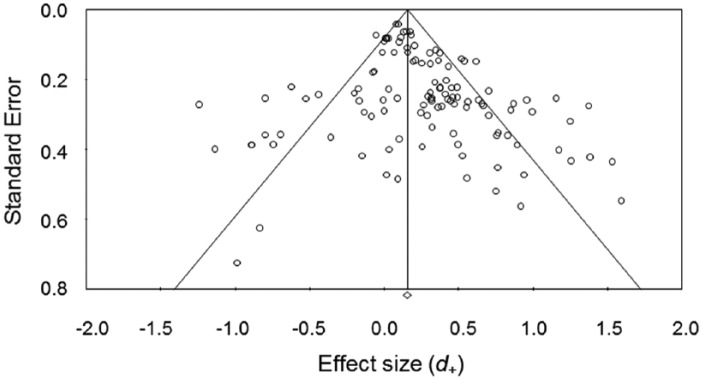
Funnel plot of effect sizes.

Funnel plots revealed that effect sizes were not symmetrically distributed, such that there was a disproportionate concentration of studies with larger effect sizes and larger standard errors (see Figure 2). Egger’s regression test (Egger et al., 1997) revealed significant asymmetry (*p* < .01). Using a random-effects model, Duval and Tweedie’s (2000) Trim and Fill method revealed evidence of publication bias, with inclusion of 18 missing studies resulting in a lower estimated effect size than the original analysis (*d*_+_ = 0.15, 95% CI = [0.08, 0.21]). However, interpretation of the adjusted effect size is similar to the unadjusted effect size, in that the effect is positive, small in magnitude, and significantly different from zero. Thus, the influence of publication bias in the current meta-analysis can be designated as modest rather than severe (Rothstein, Sutton, & Borenstein, 2005).

### Univariate Moderator Analyses

Subgroup analyses and univariate meta-regressions were conducted to examine whether the moderator variables had a significant effect on the magnitude of the QBE. Tables 1 and 2 report effect sizes and other relevant statistics for the subgroup analyses and univariate meta-regressions, respectively.

**Table 1.** Summary of Subgroup Moderator Analyses.

Moderator	*k*	*d* (95% CI)	*I* ^2^	*Q*	*p* value	Adjusted *R*^2^
Question characteristics						
Question type				6.32	.04	0.78%
Prediction	85	0.29 [0.22, 0.37]	73.82			
Mixed	16	0.14 [0.01, 0.27]	7.56			
Intention	15	0.12 [−0.04, 0.28]	41.85			
TPB items				2.11	.15	0.00%
TPB items	14	0.13 [−0.02, 0.28]	51.32			
No TPB items	102	0.26 [0.19, 0.32]	70.98			
AR items				4.90	.03	2.91%
AR items	10	0.08 [−0.07, 0.23]	0.00			
No AR items	106	0.26 [0.20, 0.33]	70.25			
Behavior characteristics						
Experience with behavior				1.30	.52	0.00%
No experience	31	0.26 [0.15, 0.38]	68.29			
Varied experience	76	0.24 [0.16, 0.32]	72.03			
High experience	9	0.14 [−0.04, 0.32]	0.00			
Behavior domain				13.78	<.01	1.59%
Health	37	0.29[0.19, 0.40]	70.93			
Consumer	21	0.34 [0.20, 0.48]	52.13			
Other	8	0.30 [0.09, 0.51]	53.06			
Prosocial	32	0.19 [0.08, 0.30]	52.06			
Risky/undesirable	18	−0.05 [−0.23, 0.13]	83.56			
Behavior frequency				0.59	.44	0.00%
One-time	68	0.26 [0.18, 0.34]	64.82			
Repeated	48	0.21[0.12, 0.30]	74.61			
Behavior measure^a^				0.80	.37	0.00%
Self-report	38	0.19 [0.08, 0.30]	78.88			
Objective	76	0.25 [0.18, 0.32]	62.51			
Methodological characteristics						
Personal contact with the experimenter				0.03	.86	0.00%
Personal	12	0.22 [0.04, 0.40]	55.91			
Impersonal	104	0.24 [0.17, 0.30]	70.95			
Research setting				9.03	<.01	13.46%
Laboratory	44	0.38 [0.28, 0.49]	58.48			
Field	72	0.17 [0.10, 0.24]	70.03			
Incentive for participation				6.41	.01	0.41%
Incentive	29	0.36 [0.25, 0.47]	66.56			
No incentive	87	0.19 [0.12, 0.26]	69.84			
Sample type				8.72	.01	10.04%
Student	76	0.31 [0.23, 0.39]	69.05			
Non-student	27	0.12 [0.02, 0.22]	52.43			
Mixed/unreported	13	0.22 [0.02, 0.42]	74.16			

*Note. k* = number of observations; *d* = standardized mean difference effect size with Hedge’s adjustment; CI = confidence intervals; *I*^2^ = Higgins and Thompson’s (2002) *I*^2^ statistic; *Q* = Cochran’s (1952) *Q* statistic; Adjusted *R*^2^ = percentage heterogeneity accounted for by the moderator. TPB = theory of planned behavior; AR = anticipated regret.

a Two studies were excluded from this moderator analysis as they used a combination of objective and self-report measures as a single outcome.

**Table 2.** Summary of Univariate Meta-Regression Moderator Analyses.

Moderator	*k*	*M* (*SD*)	*I* ^2^	Regression coefficient (95% CI)	*p* value	Adjusted *R*^2^
Mechanisms underlying the QBE						
Attitude accessibility	116	−0.43 (1.13)	69.59	0.05 [0.001, 0.10]	.047	0.00%
Attitude valence	116	3.42 (1.13)	69.28	0.002 [−0.05, 0.06]	.95	0.00%
Cognitive dissonance	116	2.39 (0.93)	69.15	−0.05 [−0.12, 0.01]	.12	1.11%
Ease of representation	116	3.23 (0.61)	67.96	−0.11 [−0.20, −0.02]	.02	2.23%
Correspondence (all studies)	116	2.34 (0.78)	69.66	−0.03 [−0.11, 0.05]	.43	0.00%
Correspondence (objective behavior measure studies only)	76	2.21 (0.81)	61.91	−0.05 [−0.13, 0.03]	.23	0.00%
Question characteristics						
Number of intention/prediction questions	116	1.32 (0.87)	68.73	−0.03 [−0.09, 0.03]	.36	1.82%
Number of behavior questions	116	6.34 (12.42)	69.20	−0.003 [−0.01, 0.001]	.11	0.00%
Behavior characteristics						
Social desirability	116	3.63 (1.27)	69.90	0.07 [0.02, 0.11]	.01	0.00%
Difficulty of behavior (linear)	116	2.35 (1.19)	68.54	−0.06 [−0.11, −0.02]	.01	3.56%
Methodological characteristics						
Time interval (ln)	116	40.08 (78.08)^a^	67.57	−0.05 [−0.08, −0.03]	<.001	7.33%
Response rate (field studies only)	72	0.86 (0.26)	70.42	0.09 [−0.15, 0.32]	.48	0.00%
Publication year	116	2004 (6.75)^b^	69.94	0.004 [−0.01, 0.01]	.34	0.00%

*Note. k* = number of observations; *I*^2^ = Higgins and Thompson’s (2002) *I*^2^ statistic; 95% CI = 95% confidence intervals; Adjusted *R*^2^ = percentage heterogeneity accounted for by the moderator; QBE = question–behavior effect.

a The mean and standard deviation of the raw time interval data, rather than log transformed data, are reported for ease of interpretation.

b The mean publication year is rounded to the nearest whole number.

#### Mechanisms underlying the QBE

There was a significant association between our index of attitude accessibility and the magnitude of the QBE; in particular, the interaction between attitude valence and response rates proved reliable (β = .05, 95% CI = [0.001, 0.10], *p* = .047). Attitude valence on its own was not reliable (β = .002, 95% CI = [−0.05, 0.06], *p* = .95). This former finding supports an attitude accessibility explanation for the QBE, although adjusted *R*^2^ indicated that this moderator explained little heterogeneity (<0.1%).

Meta-regression analysis showed no effect of cognitive dissonance (rated degree of discomfort following a prediction/behavior mismatch) on the magnitude of the QBE (β = −.05, 95% CI = [−0.12, 0.01], *p* = .12).

There was a significant negative effect of rated ease of representation on the QBE (β = −.11, 95% CI = [−0.20, −0.02], *p* = .02). However, this finding was not consistent with a behavioral simulation explanation as greater rated ease of representation was associated with *smaller* effect sizes. Calculation of adjusted *R*^2^ indicated that 2.23% of the heterogeneity was accounted for by this moderator. Meta-regression analysis revealed no significant association between QBE and correspondence between the question and behavior measures in either the full set of studies (β = −.03, 95% CI = [−0.11, 0.05], *p* = .43) or the subset of studies using objective measures of behavior (β = −.05, 95% CI = [−0.13, 0.03], *p* = .23). These findings do not support processing fluency or cognitive dissonance explanations for the QBE.

#### Question characteristics

Subgroup analysis comparing studies using intention, prediction, or mixed questions revealed that question type had a significant effect on the QBE (*Q* = 6.32, *p* = .04). Although all three question types were characterized by small positive effect sizes, studies asking participants to predict or report their expectations of future behavior had the largest effect on the QBE (*d*_+_ = 0.29, 95% CI = [0.22, 0.37]), followed by those using mixed items (*d*_+_ = 0.14, 95% CI = [0.01, 0.27]) and intention items only (*d*_+_ = 0.12, 95% CI = [−0.04, 0.28]). The effect size for intention questions was not significantly different from zero. Pairwise comparisons revealed a significant difference in effect size between studies using prediction versus mixed items (*Q* = 3.84, *p* = .05). No other comparisons were significantly different (*p*s > .10).

Subgroup analysis comparing studies measuring TPB constructs to those that did not measure TPB constructs revealed that this moderator had no significant effect on the size of the QBE (*Q* = 2.11, *p* = .15). Including questions relating to anticipated regret did have a significant effect on the QBE (*Q* = 4.90, *p* = .03). Studies that included anticipated regret items had a smaller effect size (*d*_+_ = 0.08, 95% CI = [−0.07, 0.23]) than studies that did not (*d*_+_ = 0.26, 95% CI = [0.20, 0.33]). Adjusted *R*^2^ indicated that this moderator accounted for 2.91% of the heterogeneity.

Meta-regressions showed no significant effect of the number of questions relating to behavioral prediction or intention (β = −.03, 95% CI = [−0.09, 0.03], *p* = .36), or the total number of behavior-related questions (β = −.003, 95% CI = [−0.01, 0.001], *p* = .11) on the size of the QBE, although scatterplots indicated a restricted range of scores on this measure, such that single-item measures of intention/prediction were the norm.

#### Behavior characteristics

Subgroup analysis found no effect of experience with the behavior on the magnitude of the QBE (*Q* = 1.30, *p* = .52). However, the QBE differed by behavioral domain (*Q* = 13.78, *p* < .01). Studies targeting health behaviors (*d*_+_ = 0.29, 95% CI = [0.19, 0.40]), consumer behaviors (*d*_+_ = 0.34, 95% CI = [0.20, 0.48]), and miscellaneous other behaviors (*d*_+_ = 0.30, 95% CI = [0.09, 0.51]) had small-to-medium positive effect sizes that were significantly different from zero. Studies targeting prosocial behaviors also demonstrated a significant QBE (*d*_+_ = 0.19, 95% CI = [0.08, 0.30]). However, studies targeting risky or undesirable behaviors (*d*_+_ = −0.05, 95% CI = [−0.23, 0.13]) had a small negative effect size that was not significantly different from zero. Adjusted *R*^2^ indicated that this moderator accounted for 1.59% of the heterogeneity. Pairwise comparisons revealed that the effect size for studies targeting risky behaviors was significantly smaller than studies targeting health behaviors (*Q* = 8.83, *p* < .01), consumer behaviors (*Q* = 7.31, *p* < .01), and prosocial behaviors (*Q* = 4.91, *p* = .03). Consumer behaviors also had a significantly larger effect size than prosocial behaviors (*Q* = 5.13, *p* = .02). No other two groups were significantly different (*p*s > .1), although the difference in effect size between studies targeting prosocial versus miscellaneous other behaviors approached significance (*Q* = 3.20, *p* = .07).

Social desirability and difficulty of behavior also influenced the magnitude of the QBE. More socially desirable behaviors were associated with a larger effect size (β = .07, 95% CI = [0.02, 0.11], *p* = .01). More difficult behaviors were associated with a smaller effect size in linear analyses (β = −.06, 95% CI = [−0.11, −0.02], *p* = .01), and in a multivariate model including both the linear and quadratic effects of behavior difficulty, there was also a significant quadratic association between behavioral difficulty and the QBE (β = .04, 95% CI = [0.001, 0.08], *p* = .047). Although 3.56% and 4.71% of the heterogeneity was accounted for by the behavioral difficulty moderator in linear and quadratic analyses, respectively, adjusted *R*^2^ indicated that the social desirability moderator explained little heterogeneity (<0.1%). There were no reliable effects of behavior frequency (one-time occurrence vs. repeated; *Q* = 0.35, *p* = .44), or behavior measure (self-report vs. objective; *Q* = 0.80, *p* = .37) on effect sizes.

#### Methodological characteristics

The distribution of scores for the time interval moderator was non-normal, with skewness of 2.56 (*SE* = 0.23) and kurtosis of 6.79 (*SE* = 0.45). To avoid violating assumptions of normality in meta-regression, a score of 1 was added to each time point and the resulting data were log transformed, which eliminated skewness (0.55, *SE* = 0.23) and kurtosis (−1.02, *SE* = 0.45). Meta-regression analysis of the log transformed data demonstrated a significant negative effect of time interval on the magnitude of the QBE; longer time intervals between questioning and behavior measurement were associated with smaller effect sizes (β = −.05, 95% CI = [−0.08, −0.03], *p* < .001). Examination of the adjusted *R*^2^ indicated that this moderator explained 7.33% of the heterogeneity.

Subgroup analysis showed no effect of personal contact with the experimenter (*Q* = 0.03, *p* = .86). However, the research setting moderated the QBE (*Q* = 10.83, *p* < .01); studies that measured behavior in the laboratory had a small-to-medium effect size (*d*_+_ = 0.38, 95% CI = [0.28, 0.49]), whereas studies measuring behavior in the field had a small effect size (*d*_+_ = 0.17, 95% CI = [0.10, 0.24]); 13.46% of the heterogeneity was accounted for by this moderator.

The response rate in field studies did not influence the QBE (β = .09, 95% CI = [−0.15, 0.32], *p* = .48) but the use of incentives (in all studies) did (*Q* = 6.41, *p* = .01). Studies that provided an incentive showed a small-to-medium effect size (*d*_+_ = 0.36, 95% CI = [0.25, 0.47]) where studies not providing an incentive showed a small effect size (*d*_+_ = 0.19, 95% CI = [0.12, 0.26]). However, adjusted *R*^2^ indicated that this moderator explained little heterogeneity (<0.1%).

Subgroup analyses revealed a significant effect of sample type on the QBE (*Q* = 8.72, *p* = .01), such that the QBE was the largest in studies using student samples (*d*_+_ = 0.31, 95% CI = [0.23, 0.39]), followed by mixed or unreported samples (*d*_+_ = 0.22, 95% CI = [0.02, 0.42]), followed by non-student samples (*d*_+_ = 0.12, 95% CI = [0.02, 0.22]). Pairwise comparisons revealed that the effect size for studies of student samples was significantly larger than studies targeting non-student samples (*Q* = 9.43, *p* < .01). No other two groups were significantly different (*p*s > .1). Examination of the adjusted *R*^2^ indicated that 10.04% of the heterogeneity was accounted for by this moderator.

Finally, there was no significant association between year of publication and magnitude of the QBE (β = .004, 95% CI = [−0.005, 0.01], *p* = .34).

### Relationships Between Moderators

Before undertaking multivariate analyses of the associations between effect sizes and moderator variables, we tested for collinearity among the moderators. Pearson’s correlations, point–biserial correlations, and phi-coefficients were calculated between each pair of continuous, continuous–categorical, and categorical–categorical moderators, respectively (see Table 3). The four dichotomous categorical moderators were coded as follows: TPB items (TPB = 1, no TPB = 0), anticipated regret items (anticipated regret [AR] = 1, no AR = 0), research setting (laboratory = 1, field = 0), and incentive for participation (incentive = 1, no incentive = 0). The three categorical moderators that had more than two levels (question type, behavior domain, and sample type) were dummy coded into dichotomous variables as follows: question type (prediction = 1, other = 0), behavior domain (risky/undesirable behaviors = 1, other = 0), and sample type (student sample = 1, other = 0).

**Table 3.** Correlations Between All Moderators.

Moderator	1	2	3	4	5	6	7	8	9	10	11	12	13	14	15	16	17	18	19	20	21	22	23
1. Attitude accessibility	—																						
2. Attitude valence	−.11	—																					
3. Cognitive dissonance	−.20*	.31***	—																				
4. Ease of representation	−.16	−.01	.01	—																			
5. Correspondence	−.10	.09	.13	.10	—																		
6. Q type	.46***	−.31***	−.24**	−.28**	−.06	—																	
7. TPB items	−.35***	.41***	.39***	.11	−.07	−.49***	—																
8. Anticipated regret items	−.43***	.32***	.28**	.24*	−.06	−.51***	.72***	—															
9. Number of intention/prediction Qs^a^	−.26**	.15	.28**	.15	−.14	−.29**	.42***	.52***	—														
10. Number of behavior-related Qs^b^	−.01	.24**	.22*	.19*	.05	−.48***	.56***	.54***	.40***	—													
11. Experience with behavior	.14	.14	.18	.04	.09	.13	−.10	−.19*	−.10	−.08	—												
12. Behavior domain	−.05	−.57***	.02	.19*	.12	.26**	−.18*	−.13	−.16	−.19*	.24*	—											
13. Social desirability	.003	.66***	.36***	−.27**	.10	−.24**	.24**	.16	.09	.25**	−.05	−.72***	—										
14. Difficulty of behavior	−.52***	.40***	.54***	.05	.12	−.48***	.54***	.48***	.33***	.26**	.11	−.05	.25**	—									
15. Behavior frequency	−.15	.16	.05	.28**	.28**	−.17	−.02	.18	.01	.03	.17	.12	−.13	.09	—								
16. Behavior measure	.10	−.02	.06	.13	.26**	−.08	−.15	−.15	−.21*	−.05	.21*	.31***	−.16	.09	.53***	—							
17. Time interval	−.46***	.31***	.32***	.30**	.23*	−.60***	.39***	.43***	.28**	.32***	.11	−.005	.06	.59***	.47***	.30**	—						
18. Personal contact with the experimenter	.14	−.004	.02	.06	.14	.08	−.15	−.10	−.06	−.11	.05	−.15	.21*	.04	−.06	−.24*	.14	—					
19. Research setting	.24*	−.22*	−.42***	−.21*	−.42***	.39***	−.14	−.24**	−.29**	−.17	−.12	.01	−.12	−.43***	−.51***	−.43***	−.72***	−.27**	—				
20. Response rate	.84***	−.43***	−.31***	−.12	−.21*	.63***	−.41***	−.43***	−.26**	−.10	−.004	.18	−.26**	−.59***	−.14	.08	−.51***	.14	.33***	—			
21. Incentive for participation	−.03	−.12	.15	−.06	−.03	.12	−.08	−.04	−.05	−.01	−.01	.03	−.004	−.12	−.12	−.23*	−.30**	−.20*	.29**	.08	—		
22. Sample type	.18	−.11	−.08	−.08	.07	.30**	−.39***	−.42***	−.30**	−.36***	.24**	.11	−.08	−.30***	.20*	.27**	−.39***	−.29**	.12	.14	.04	—	
23. Publication year	−.26**	.002	.02	.11	−.02	−.18*	.12	.29**	.21*	.07	−.08	.06	−.08	−.02	.27**	.09	−.03	−.46***	.08	−.24*	.04	.07	—

*Note. N* = 116 for all analyses. TPB = theory of planned behavior; Q = question.

a Number of intention/prediction questions.

b Number of behavior-related questions.

* *p* < .05. **p < .01. ***p < .001.

Table 3 shows that several moderators were significantly intercorrelated. To address excessive collinearity between some pairs of moderators (*r*s > .70) while retaining information from all moderators, we combined three pairs of highly correlated predictors into single measures (time and research setting; behavioral domain and social desirability; presence of questions assessing TPB constructs and presence of questions assessing anticipated regret) before conducting multivariate analyses. Time interval and research setting were highly correlated (*r* = .72, *p* < .001), such that 91% of studies in a lab setting measured behavior immediately after questioning, compared with only 4% of studies in a field setting. Calculating a mean score was not appropriate in this case given the different measurement scales, so we generated a regression factor score to allow us to retain information from both variables, with scores indicating the location of each study on this factor (DiStefano, Zhu, & Mindrila, 2009). Higher scores indicate greater use of field settings and longer time intervals. Similarly, we calculated a factor score representing behavior domain and social desirability, two aspects of the behavior that were highly correlated (*r* = −.72, *p* < .001). Risky behaviors tended to also be low in social desirability (*M* social desirability = 1.50, *SD* = 0.86), whereas non-risky behaviors tended to be high in social desirability (*M* social desirability = 4.00, *SD* = 0.88); thus, higher scores on this factor indicate behaviors that are less risky and higher in social desirability. Finally, studies that included questions on TPB constructs were highly likely to also include questions assessing anticipated regret (*r* = .72, *p* < .001). To combine these moderators, we computed a combined score ranging from zero to two, to indicate whether studies had assessed neither of these constructs, one of these constructs, or both of these constructs.

### Multivariate Meta-Regression

**Figure 3.**
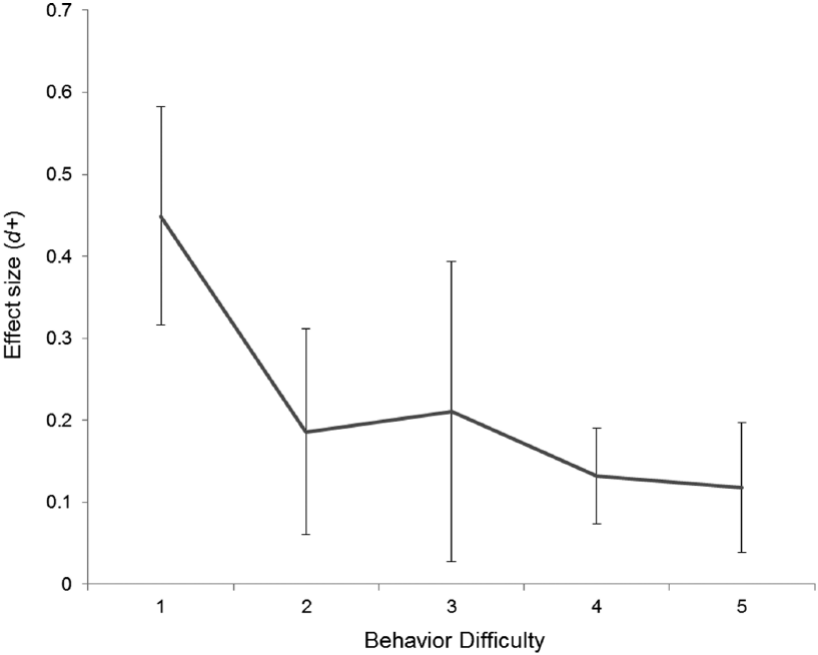
Weighted effect size (*d*+) at each level of behavior difficulty. *Note.* Error bars indicate 95% confidence intervals.

We constructed an overall multivariate model by including all of the predictors that were significantly associated with the QBE in univariate analyses (i.e., attitude accessibility, ease of representation, question type, a combined score for the presence of TPB and anticipated regret questions, the factor score for behavior domain and social desirability, the linear and quadratic effects of difficulty of behavior, the factor score for time interval and research setting, incentive for participation, and sample type). In this model, the factor score for behavior domain and social desirability (β = .17, 95% CI = [0.08, 0.26], *p* < .001), the quadratic effect of difficulty of behavior (β = .05, 95% CI = [0.002, 0.10], *p* = .04, see Figure 3), and sample type (β = .15, 95% CI = [0.004, 0.30], *p* = .04), each predicted unique variance in the QBE; none of the other moderators predicted unique variance (*p*s > .12; see Table 4).

**Table 4.** Multivariate Meta-Regression Analyses.

Moderator variables	Regression coefficient (95% CI)	*p* value	Adjusted *R*^2^
Overall model			0.00%
Attitude accessibility	0.05 [−0.03, 0.13]	.19	
Ease of representation	0.01 [−0.10, 0.11]	.92	
Question type^a^	0.07 [−0.13, 0.28]	.47	
TPB and AR items^b^	−0.04 [−0.17, 0.09]	.55	
Behavior domain and social desirability^c^	0.17 [0.08, 0.26]	<.001	
Difficulty of behavior (linear)	−0.06 [−0.15, 0.03]	.17	
Difficulty of behavior (quadratic)	0.05 [0.002, 0.10]	.04	
Time interval and research setting^d^	0.004 [−0.11, 0.12]	.94	
Incentive for participation^e^	0.12 [−0.03, 0.27]	.12	
Sample type^f^	0.15 [0.004, 0.30]	.04	

*Note.* 95% CI = 95% confidence intervals; TPB = theory of planned behavior; AR = anticipated regret.

a Question type was coded prediction = 1, other = 0.

b TPB and AR questions were coded 0 = of these items present, 1 = one of these items present, 2 = both of these items present.

c Factor score for behavior domain and social desirability; higher scores indicate behaviors that are less risky and higher in social desirability.

d Factor score for time interval and research setting; higher scores indicate longer time intervals and greater use of field settings.

e Incentive for participation was coded incentive = 1, no incentive = 0.

f Sample type was coded student sample = 1, other = 0.

## Discussion

The current meta-analysis aimed to provide a comprehensive quantitative review of the impact of asking self-prediction and intention questions on a broad range of behaviors, and shed light on the proposed mediators and moderators of this QBE. A random-effects model indicated that prediction/intention questions have a small positive effect on subsequent behavior. This effect size is consistent with Spangenberg and Greenwald’s (1999) previous meta-analysis on the QBE. In addition, the effect size was characterized by significant heterogeneity, suggesting the presence of moderating variables, which we examined using subgroup analyses and meta-regressions. Univariate analyses concerning theoretical mechanisms underlying the QBE revealed significant moderating effects of attitude accessibility and ease of representation, but no significant effects for rated cognitive dissonance or measurement correspondence (in either the full set of studies or subset of studies using objective measure of behavior). Univariate moderator analysis concerning characteristics of the question, behavior, and methodology revealed that the QBE was stronger when prediction rather than intention questions were asked, when anticipated regret questions were not asked, when the behavior domain was low-risk, when the behavior was more socially desirable and less difficult, when the time interval between questioning and behavior was short, when the research was conducted in the lab, and when incentives and student participants were used. There were no significant effects for use of questions based on the TPB, the number of intention or prediction questions, the number of behavior-related questions, experience with the behavior, behavior frequency, type of behavior measure, personal contact with the experimenter, the response rate to the questions, or publication year. Multivariate moderator analyses indicated that the combined score for the behavior domain and social desirability of the behavior, the quadratic term for difficulty of behavior, and the sample type were the only predictors that explained unique variance in the QBE.

### Mechanisms Underlying the QBE

The current meta-analysis attempted to evaluate three key mechanisms proposed to underlie the QBE—the attitude accessibility, cognitive dissonance, and behavior simulation/processing fluency accounts—by examining the impact of theoretically based moderators on the magnitude of the QBE. The attitude accessibility account of the QBE proposes that the strength of the QBE depends on both the valence of attitudes and the proportion of the sample whose attitudes are activated (i.e., the response rate to questioning). Univariate analysis revealed a significant interaction between attitude valence and response rate, such that greater activation of more positive attitudes was associated with larger effect sizes. Although this finding is consistent with an attitude accessibility account of the QBE, multivariate analyses indicated that attitude accessibility did not predict unique variance in effect sizes when the other moderators were included in the meta-regression. Moreover, there were several findings that are not easily accounted for by the attitude accessibility explanation of the QBE. For instance, whereas attitude accessibility explanations of QBE would predict significant effects for the number of behavioral prediction or intention items, total number of items relating to behavior, and experience with the behavior, there were no reliable effects of these moderators in the current meta-analysis. The univariate analyses also indicated that question type moderated the size of the QBE, such that prediction questions had a larger effect on behavior than intention questions. However, there is no accessibility-based reasoning to suggest that prediction questions should prompt greater accessibility of attitudes than intention questions (see Wood et al., 2014).

We observed little support for the cognitive dissonance explanation of the QBE. Rated dissonance was not associated with effect sizes in univariate analyses. Other moderator effects also did not fit neatly with a cognitive dissonance account. For instance, consumer behaviors had a significantly larger effect size than prosocial behaviors but seem less likely to be associated with strong normative or personal standards, or invoke the same degree of discomfort when confronted with a discrepancy between predictions or intentions and past behavior. Similarly, although answering multiple questions about predictions of future behavior should increase dissonance, there was no significant effect of number of intention/prediction questions on the QBE.

The behavioral simulation and processing fluency explanations of the QBE also did not find support in the present meta-analysis. Although processing fluency accounts would predict that measurement correspondence influences the magnitude of the QBE, no significant effect of this moderator was observed, either in the full sample of studies or in the subset of studies using objective measures of behavior. In addition, although there was a significant association between ease of representation and the QBE in the univariate analyses, the direction of this effect was inconsistent with a behavioral simulation explanation. That is, greater rated ease of representation was associated with *smaller* rather than larger effect sizes. Moreover, multivariate analyses indicated that ease of representation did not predict unique variance in the QBE, over and above the other moderators.

### Characteristics of the Question, Behavior, and Methodology as Moderators of the QBE

The present meta-analysis found that several characteristics of the question, behavior, and methodology moderated the magnitude of the QBE in univariate analyses. First, the type of question used in QBE interventions influenced effect sizes. Specifically, self-prediction questions promoted behavioral performance, whereas there was no significant QBE for intention questions. The superior impact of self-predictions compared with intention questions might be expected on the basis of Sheppard et al.’s (1988) analysis of the role of desirability versus feasibility considerations in determining expectations compared with intentions. Whereas intentions are based more on desirability than feasibility, self-predictions are based on both feasibility and desirability, and self-predictions better predict subsequent behavior compared with intentions (Sheppard et al., 1988). At the same time, however, it is important to note that studies that used intention questions were all field studies and most had relatively long intervals between questioning and behavior—both attributes that were associated with smaller effect sizes. This finding suggests that further tests of question type in field settings over longer time intervals would be valuable.

Second, univariate analyses indicated that including questions relating to the TPB had no effect on the magnitude of the QBE. We reasoned that activating behavioral, normative, and control considerations alongside reporting one’s intentions could either increase or decrease the QBE, but we observed no association with effect sizes. Questions relating to anticipated regret, however, did have a significant effect on the QBE. Anticipated regret questions were expected to increase the magnitude of the QBE, as the potentially aversive consequences of not acting can strengthen intentions to act (Abraham & Sheeran, 2003; Godin et al., 2010; Sandberg & Conner, 2009). However, the effect observed here was in the opposite direction—studies that included anticipated regret questions had *smaller* effect sizes than studies that did not. One possible explanation of this finding is that anticipated regret items are seen as manipulative by participants and thus promote reactance (Godin et al., 2010). Further primary research is needed to gauge reactions to anticipated regret questions and to investigate the circumstances in which these questions are resisted.

Third, neither behavioral frequency (repeated vs. one-time performance) nor the type of behavior measure used (self-report vs. objective) was a significant moderator variable. The former finding speaks to the generality of the QBE, whereas the latter finding rules out the idea that the QBE is merely due to biased self-reported behavior. Univariate analysis demonstrated a significant negative association between time interval and the QBE, such that studies with a longer period between behavioral prediction and measurement of behavior were characterized by smaller effect sizes than studies with a shorter time interval. This temporal decline in the magnitude of the QBE has practical implications as it suggests that “top-up” QBE treatments or additional techniques may be needed to promote long-term changes in behavior. The magnitude of the QBE was also affected by the research setting. In particular, laboratory-based measures of behavior had a significantly larger effect size than measures from the field. On one hand, it is possible that laboratory-based measures are more accurate than field measures. On the other hand, laboratory studies could generate greater experimenter demand, such that participants feel pressure to enact their intentions or predictions because of the authority of the setting. Studies that measure the same behavior in different settings are needed to unravel this issue.

Fourth, provision of an incentive led to a larger effect of prediction/intentions questions on behavior. We speculated that incentives could increase the QBE by improving response rates. However, there was no significant association between incentive provision and response rate; in fact, response rate was not associated with the QBE in field studies. There is a large and complex literature concerning the impact of incentives on task performance (see, for example, Bonner, Hastie, Sprinkle, & Young, 2000; Camerer & Hogarth, 1999, for reviews). In general, provision of an incentive appears to improve behavioral performance at least when (a) effort is needed to perform the behavior, (b) the behavior does not require specific skills, and (c) there is little intrinsic motivation to undertake the behavior. Research on incentive effects appears to be mute concerning other possibilities that are relevant in the context of the QBE, however. For instance, incentives could increase the motive to be consistent or make participants feel accountable to the experimenter (Lerner & Tetlock, 1999) and so strengthen relations between predictions/intentions and behavior. We did not observe a significant interaction between rated dissonance and incentive provision, which would seem to suggest that incentives do not increase consistency motives. However, it remains possible that the impact of incentives on the magnitude of the QBE is explained, at least in part, by accountability concerns. Further research is needed to examine whether incentive provision exerts its impact because enacting the predicted or intended behavior is expected to lead to reward, because participants feel more accountable for acting on their predictions/intentions, or both.

The significant moderator effects observed for attitude accessibility, ease of representation, question type, use of anticipated regret questions, time interval, research setting, and provision of an incentive in univariate analyses were no longer reliable when all moderator variables were considered simultaneously in a multivariate meta-regression. Only three moderator variables captured unique variance in effect sizes, namely, the factor score for behavior domain/social desirability, the quadratic effect of behavioral difficulty, and sample type. Some researchers have argued that the QBE is likely to prompt increases in socially normative behaviors only (Spangenberg & Greenwald, 1999), whereas others have expressed concern that the QBE could exacerbate performance of socially undesirable or risky behaviors (Fitzsimons & Moore, 2008a). The findings obtained here are more consistent with the former viewpoint. In particular, whereas studies targeting health, consumer, prosocial, and miscellaneous behaviors all showed significant, small-to-medium effect sizes in the univariate analyses, studies of risky or undesirable behaviors were characterized by a small negative effect size that was not significantly different from zero. The association between rated social desirability and the magnitude of the QBE was also reliable in univariate analyses. The present analyses may therefore go some way toward allaying concerns about a detrimental impact of the QBE.

The quadratic association between ratings of behavioral difficulty and QBE was reliable in the multivariate meta-regression. The relationship between behavioral difficulty and behavioral performance is complex. In some studies, a positive relationship is observed such that people perform better on difficult goals (e.g., Locke, 1968; Locke, Shaw, Saari, & Latham, 1981); in other studies, greater perceived difficulty is associated with reduced behavioral performance (see, for example, Armitage & Conner, 2001; McEachan, Conner, Taylor, & Lawton, 2011, for meta-analyses), whereas Atkinson (1966) proposed an inverted U relationship such that goals of medium difficulty generate greater motivation and performance compared with both easy and difficult goals. In the present research, there was a negative curvilinear relationship between difficulty and the QBE; at the lowest rating of difficulty, the QBE was of medium magnitude, whereas the QBE was small at every other level of difficulty (see Figure 3). This finding indicates that behavioral difficulty influences the magnitude of the QBE but does not indicate what features of difficulty drive this association. Primary research directed at understanding the nature of the resources, opportunity, time, skills, or effort that could serve to enhance the QBE for particular behaviors would be valuable.

There was a linear relationship between sample type and the QBE in the multivariate analysis such that the greater the student composition of the sample, the larger the effect. Henrich et al. (2010) provided an extensive review of differences between students and non-student adults. It is known that students are more prone to dissonance effects, exhibit higher degrees of self-monitoring, and show greater susceptibility to attitude change and social influence compared with their counterparts. However, it is not yet clear which of these features (or combination of features) may account for the impact of sample type on the QBE.

### Limitations and Future Directions

The first limitation of the present meta-analysis is the paucity of studies that could be included in the review. Although the review started with more than 3,000 records, only a limited number of tests were available to assess key hypotheses and to examine the impact of particular moderator variables within levels of other moderator variables (e.g., the impact of question type for non-student samples). A second limitation concerned our reliance on observer ratings to code key variables (e.g., the likely discomfort that would accrue from non-performance of the behavior, experience with the behavior). Ideally, scores on these variables would be synthesized from the original research articles. However, observer ratings were used here because, almost invariably, these variables were not measured in the original studies. Although the independent ratings were reliable and permitted tests of key hypotheses, we acknowledge that the validity of these ratings is indeterminate, and so caution is warranted in drawing conclusions from moderator analyses that involved observer ratings. Future tests of the cognitive dissonance explanation for the QBE should deploy direct measures of dissonance, such as participant ratings of discomfort (e.g., Elliot & Devine, 1994) or physiological measures of skin conductance response (e.g., Harmon-Jones, Brehm, Greenberg, Simon, & Nelson, 1996). Given the potential for self-report measures of dissonance to affect the QBE manipulation itself, use of physiological measures may be preferable. Similar caution is also warranted in drawing conclusions on the basis of the indirect measure of attitude accessibility used here. Few studies included process measures of accessibility, and it was necessary to use an alternative and rather broad index of accessibility here. Whenever possible, direct measures of attitude accessibility, cognitive dissonance, and experience with the behavior should be deployed in future studies.

A third limitation of the present review is the relatively high level of heterogeneity observed in the effect sizes from the primary studies (*I*^2^ = 69.70%). We anticipated that the effects would be heterogeneous and therefore tested numerous conceptual and methodological moderators. It should be acknowledged, however, that the proportion of heterogeneity explained by moderator variables generally was extremely modest here (<1% for the significant moderators in the multivariate analyses). These findings indicate that additional moderators of the QBE remain to be identified and suggest that further primary research studies are needed to determine the boundary conditions of the effect.

Perhaps the greatest limitation of the current meta-analysis accrues from the high degree of multicollinearity among moderators inherent in the QBE literature. Dholakia (2010) and others have noted that QBE research encompasses relatively distinct “clusters,” which tend to share a common methodology and theoretical moderators. For example, Sprott, Spangenberg, and Block, et al. (2006) pointed out that “mere-measurement” studies tend to use intention questions and focus on a variety of different behaviors, whereas “self-prophecy” studies rely on prediction questions and focus on socially normative behaviors. Similarly, questions targeting TPB constructs and anticipated regret are exclusively found in studies targeting health or prosocial behaviors, whereas the vast majority of studies conducted in laboratory-based settings use a single, prediction question. The current meta-analysis found that many significant moderators exhibited moderate to large intercorrelations. This finding has two key implications. First, researchers interested in the effect of intention/prediction questions on behavior should seek to unite, and go beyond, the theoretical and methodological traditions of their respective clusters to systematically test the moderators enumerated here (cf. Sprott, Spangenberg, Block, et al., 2006). Second, although our efforts to disentangle the unique variance accounted for by many different moderator variables using multivariate meta-regression are a strength of the present work, a key purpose of meta-analysis is to identify pressing issues for future primary research (Peters, de Bruin, & Crutzen, 2015). As Peters et al. noted, meta-analysis should be followed by primary research that systematically tests moderator variables in fully factorial designs, to form an iterative evidence base. The present meta-analysis offers valuable clues about the moderator variables that can and should be tested in future studies to better understand and optimize applications of the QBE.

We suggest two key priorities for future research. First, to advance our understanding of the mechanisms underlying the QBE, further research is needed that directly pits the cognitive dissonance, attitude accessibility, and behavioral simulation/processing fluency explanations for the QBE against one another. It will be important that either direct measures of cognitive dissonance (e.g., skin conductance response, Harmon-Jones et al., 1996), attitude accessibility (e.g., response latency measures, Wood et al., 2014), and processing fluency are used so that mediation analyses can be undertaken, or that studies follow sequentially to establish the causal chain (Spencer, Zanna, & Fong, 2005). Research may need to be programmatic as it remains to be determined whether different mechanisms operate depending on characteristics of the behavior, questions, or sample.

Second, studies are needed to determine when the QBE is effective in field settings, and how the effect can best be harnessed to promote “real-world” behavior change. For example, it is unclear why the QBE was effective in increasing frequency of blood donation in Godin et al.’s (2010; Godin et al., 2008) studies, whereas a similar intervention by van Dongen, Abraham, Ruiter, and Veldhuizen (2013) observed no effect. What can be done to effectively exploit the QBE in efforts to increase the supply of blood? This line of work is important because although the present meta-analysis indicates that field trials of the QBE that involve non-student adult participants and no incentive have small effect sizes (.10 < *d* < .20), even effects that are small in conventional terms can be hugely valuable in social policy and public health terms. This is because QBE interventions have the potential to be both wide-reaching and very low-cost compared with other, more intensive approaches.

## Conclusion

The present findings suggest that the QBE is effective in promoting a wide range of behaviors, regardless of participants’ experience with the behavior, whether the behavior is one-off or repeated, or measured objectively or through self-report. Asking prediction or intention questions has the greatest impact on action when the behavior is socially desirable or does not involve risky behavior, when the behavior is easy to perform, and when students are the participants. We observed little support for the attitude accessibility, cognitive dissonance, or behavioral simulation and processing fluency explanations of the QBE across the studies examined here. Direct, comparative tests of these and alternative mechanisms are needed, together with field studies geared at optimizing the QBE’s potential for behavior change. Although QBE research has in the past followed distinct paths, we echo previous calls for these paths to merge to better understand and reap the benefits of this important phenomenon.

## Supplementary Material

Supplementary material

## Supplementary Material

Supplementary material

## Notes

1. A search of the Web of Science database using the terms *measurement reactivity* and *questionnaire effects on behavior* generated 12,245 and 8,066 matches, respectively. These searches were subsequently narrowed to the psychology research area only.

2. Although we excluded any experimental conditions that included implementation intentions, the control group used by Fitzsimons, Nunes, and Williams (2007) for each of two target behaviors comprised the prediction-only condition, the prediction plus implementation intentions, and the prediction plus self-reward condition for the alternative behavior.

3. We also conducted analyses using an alternative technique for dealing with non-independence of data. In cases of non-independence resulting from reporting multiple methods of analysis or data screening, this analysis still included only the most conservative means of analysis that excluded the least participants, as per the original analysis. However, in all other cases of non-independence, an alternative strategy of calculating an average effect size that collapses over multiple non-independent observations (as suggested by Higgins & Green, 2011, Section 16.5.4) was used, such that 88 tests of the question–behavior effect (QBE) were included in total. In cases where the collapsed observation crossed levels of a specific categorical moderator, this observation was necessarily excluded from that analysis. In cases where the collapsed observation encompassed multiple values of a continuous moderator, a mean score was calculated. The results of this alternative analysis mirrored the original analysis with a small significant positive effect of 0.26\ (95% confidence interval [CI] = [0.20, 0.32]), and a significant moderate-to-high degree of heterogeneity in effect sizes (*I*^2^ = 66.47%; *Q* = 259.44, *p* < .001). Moderator effects in the univariate analyses were generally less significant, but the size and direction of the effects remained largely comparable. Given the reduction in power, and loss of key moderator data inherent in using this alternative strategy, the resulting changes in statistical significance are not unexpected. Key differences in significance were that the previously significant effects of attitude accessibility (β = .04, 95% CI = [−0.01, 0.09], *p* = .13), social desirability (β = .04, 95% CI = [−0.01, 0.09], *p* = .15), rated ease of representation (β = −.061, 95% CI = [−0.16, −0.03], *p* = .20), anticipated regret (*Q* = 2.77, *p* = .10), and behavior domain (*Q* = 5.59, *p* = .23) on the QBE became non-significant, and the previously significant effect of question type became marginally significant (*Q* = 5.197, *p* = .07). In addition, the previously non-significant effect of cognitive dissonance became marginally significant (β = −.06, 95% CI = [−0.13, 0.003], *p* = .06). Consistent with the univariate findings, two of three results in the final multivariate model became non-significant (combined score for behavior domain and social desirability, β = −.08, 95% CI = [−0.27, 0.11], *p* = .40; sample type, β = .13, 95% CI = [−0.03, 0.28], *p* = .11), but the size and direction of the other effect remained comparable (quadratic effect of behavior difficulty, β = .05, 95% CI = [0.003, 0.11], *p* = .04).
