# Supplementary material for: The Impact of Asking Intention or Self-Prediction Questions on Subsequent Behavior: A Meta-Analysis
Source: Pers Soc Psychol Rev. 2015 Jul 10;20(3):245–68. doi: 10.1177/1088868315592334 (PMC4931712; doi:10.1177/1088868315592334)
Supplement: Supplementary material [file 10.1177_1088868315592334_AppendixA.pdf]

Appendix A

Effect Sizes, Study Descriptions, and Moderator Coding of Mechanisms for Studies Included in the Meta-Analysis.

Author(s) and publication year	Study (comparison)[a]	Comparison description	N[b]	*d* [c]	SE (adjusted)[d]	Mechanisms				
						Att. Acc.[e]	Att. Val.[f]	Cog. Diss. [g]	Ease of Rep.[h]	Corres.[i]
Ayres et al. (2013)	1	Compares measurement of intention/prediction to control cholesterol through diet vs. no measurement of intention/prediction, in participants given no heart age feedback, on choice to receive health plan	146	0.03	0.23	0.21	4	4.00	3	0
Chandon et al. (2004)	1(1)	Compares measurement of intention/prediction to purchase groceries again from the target company vs. no measurement of intention/prediction, on repeat purchase incidence up to 9 months after measurement	391	0.19	0.15	-0.16	3	1.00	5	2
Chandon et al. (2004)	1(2)	Compares measurement of intention/prediction to purchase groceries against from the target company vs. no measurement of intention/prediction, on repeat purchase incidence up to 2 months after measurement	391	0.34	0.21	-0.16	3	1.67	5	2
Chandon et al. (2011)	1(1)	Compares measurement of prediction to exercise vs. measurement of prediction to read/watch the news, on time spent exercising	50	-0.13	0.29	0.21	4	3.67	3	3
Chandon et al. (2011)	1(2)	Compares measurement of prediction to read/watch the news vs. measurement of prediction to exercise, on time spent reading/watching the news	50	0.25	0.29	0.21	4	1.67	3	3
Chapman (2001)	2(1)	Compares multiple measurement of predicted likelihood of returning questionnaire vs. no measurement of predicted likelihood of returning questionnaire, on return incidence after 1 day	436	0.45	0.22	-0.16	3	3.00	3	1
Chapman (2001)	2(2)	Compares multiple measurement of predicted likelihood of returning questionnaire vs. no measurement of prediction of predicted likelihood of returning questionnaire, on return incidence after 6 days	436	0.45	0.26	-0.16	3	2.67	3	1
Chapman (2001)	2(3)	Compares single measurement of predicted likelihood of returning questionnaire vs. no measurement of predicted likelihood of returning questionnaire, on return incidence after 1 day	469	0.38	0.22	-0.16	3	2.33	3	1
Chapman (2001)	2(4)	Compares single measurement of predicted likelihood of returning questionnaire vs. no measurement of predicted likelihood of returning questionnaire, on return incidence after 6 days	469	0.32	0.26	-0.16	3	2.00	3	1
Cioffi & Garner (1998)	1(1)	Compares measurement of intention to donate during the summer blood drive in the active no response condition vs. no measurement of intention to donate in the no message condition, on blood donation	373	0.49	0.39	-1.46	4	1.33	2	4
Cioffi & Garner (1998)	1(2)	Compares measurement of intention to donate during the summer blood drive in the active yes response condition vs. no measurement of intention to donate in the no message condition, on blood donation	371	0.25	0.39	-1.70	4	3.33	4	4
Cioffi & Garner (1998)	1(3)	Compares measurement of intention to donate during the summer blood drive in the forced choice response condition vs. no measurement of intention to donate in the no message condition, on blood donation	376	0.03	0.40	-0.64	4	3.33	3	4

Author(s) and publication year	Study (comparison)[a]	Comparison description	N[b]	*d* [c]	SE (adjusted)[d]	Mechanisms				
						Att. Acc.[e]	Att. Val.[f]	Cog. Diss. [g]	Ease of Rep.[h]	Corres.[i]
Conner et al. (2011)	1	Compares measurement of intention/prediction to attend a health check vs. no measurement of intention/prediction, on health check attendance	384	0.35	0.12	-1.04	4	3.33	3	3
Conner et al. (2011)	2	Compares measurement of intention/prediction to get vaccinated against flu vs. no measurement of intention/prediction, on uptake of flu vaccination	1200	0.13	0.07	0.35	3	3.33	3	3
Cox et al. (2012)	1(1)	Compares measurement of prediction to get vaccinated against Hepatitis B virus vs. no measurement of prediction, in high barrier patients, on uptake of Hepatitis B virus vaccination	262	0.52	0.14	-0.16	3	3.67	2	3
Cox et al. (2012)	1(2)	Compares measurement of prediction to get vaccinated against Hepatitis B virus vs. no measurement of prediction, in low barrier patients, on uptake of Hepatitis B virus vaccination	913	-0.06	0.07	-0.16	3	4.33	4	3
Fitzsimons & Williams (2000)	1(1)	Compares measurement of predicted likelihood of trying a New Zealand candy bar vs. measurement of predicted likelihood of trying a Canadian fruit punch, in the divided attention/magnified condition, on choice of target candy	*70*	0.47	0.27	-0.53	2	1.33	3	2
Fitzsimons & Williams (2000)	1(2)	Compares measurement of predicted likelihood of trying a New Zealand candy bar vs. measurement of predicted likelihood of trying a Canadian fruit punch, in the divided attention,/reduced condition, on choice of target candy	*70*	0.39	0.28	-0.53	2	1.00	3	2
Fitzsimons & Williams (2000)	1(3)	Compares measurement of predicted likelihood of trying a New Zealand candy bar vs. measurement of predicted likelihood of trying a Canadian fruit punch, in the full attention/magnified condition, on choice of target candy	*70*	0.67	0.28	-0.53	2	1.33	3	2
Fitzsimons & Williams (2000)	1(4)	Comparing measurement of predicted likelihood of trying a New Zealand candy bar vs. measurement of predicted likelihood of trying a Canadian fruit punch, in the full attention/reduced condition, on choice of target candy	*70*	0.36	0.28	-0.53	2	1.00	3	2
Fitzsimons & Williams (2000)	2(1)	Compares measurement of predicted likelihood of volunteering vs. no measurement of predicted likelihood, in the divided attention/magnified condition, on choice of target charity	*76*	0.56	0.26	0.59	5	2.33	3	2
Fitzsimons & Williams (2000)	2(2)	Compares measurement of predicted likelihood of volunteering vs. no measurement of predicted likelihood, in the divided attention/reduced condition, on choice of target charity	*76*	0.43	0.26	0.59	5	2.00	3	2
Fitzsimons & Williams (2000)	2(3)	Compares measurement of predicted likelihood of volunteering vs. no measurement of predicted likelihood, in the full attention/magnified condition, on choice of target charity	*76*	0.99	0.29	0.59	5	3.33	3	2
Fitzsimons & Williams (2000)	2(4)	Compares measurement of predicted likelihood of volunteering vs. no measurement of predicted likelihood, in the full attention/reduced condition, on choice of target charity	*76*	0.31	0.26	0.59	5	3.00	3	2
Fitzsimons et al. (2007)	1	Compares measurement of predicted number of classes missed vs. no measurement of prediction, on number of missed classes	81	0.49	0.22	-0.53	2	3.67	3	4
Fitzsimons et al. (2007)	4(1)	Compares measurement of predicted number of times participants would go out drinking vs. measurement of predicted number of times participants would watch TV instead of studying, on number of times that had consumed more than two drinks in a sitting	*81*	1.37	0.28	-0.53	2	2.33	4	3

Author(s) and publication year	Study (comparison)[a]	Comparison description	N[b]	*d* [c]	SE (adjusted)[d]	Mechanisms				
						Att. Acc.[e]	Att. Val.[f]	Cog. Diss. [g]	Ease of Rep.[h]	Corres.[i]
Fitzsimons et al. (2007)	4(2)	Compares measurement of predicted number of times participants would watch TV instead of studying vs. measurement of predicted number of times they would go out drinking, on number of times participants had watched television instead of studying	*81*	0.63	0.26	-0.16	3	3.67	4	3
Godin et al. (2008)	1(1)	Compares measures of intention/prediction to give blood vs. no measurement of intention/prediction, on number of registrations for blood donation within 12 months after measurement	4672	0.08	0.04	-0.56	5	2.67	5	2
Godin et al. (2008)	1(2)	Compares measures of intention/prediction to give blood vs. no measurement of intention/prediction, on number of registrations for blood donation within 6 months after measurement	4672	0.09	0.04	-0.56	5	2.67	5	3
Godin et al. (2010)	1(1)	Compares measurement of intention/prediction to give blood and anticipated regret vs. no measurement of intention/prediction, on number of registrations for blood donation within 12 months after measurement	1767	0.02	0.08	-3.37	5	3.00	4	2
Godin et al. (2010)	1(2)	Compares measurement of intention/prediction to give blood and anticipated regret vs. no measurement of intention/prediction, on number of registrations for blood donation within 6 months after measurement	1767	0.03	0.08	-3.37	5	3.00	4	3
Godin et al. (2010)	1(3)	Compares measurement of intention/prediction to give blood vs. no measurement of intention/prediction, on number of registrations for blood donation within 12 months after measurement	1753	0.01	0.08	-3.23	5	3.00	4	2
Godin et al. (2010)	1(4)	Compares measurement of intention/prediction to give blood vs. no measurement of intention/prediction, on number of registrations for blood donation within 6 months after measurement	1753	0.01	0.08	-3.23	5	3.00	4	3
Godin et al. (2011)	1	Compares measurement of intention/prediction to participate in physical activity vs. no measurement of intention/prediction, on levels of physical activity	374	0.20	0.10	0.21	4	3.67	2	1
Greenwald et al. (1987)	1	Compares measurement of expectation of registering to vote vs. no measurement of expectation, on voting registration	62	0.53	0.42	-0.16	3	2.00	3	3
Greenwald et al. (1987)	2	Compares measurement of expectation to vote vs. no measurement of expectation, on voting turnout	60	0.77	0.35	-0.16	3	2.67	3	3
Janiszewski & Chandon (2007)	4(1)	Compares measurement of plans to purchase the second-favourite candy bar vs. no measurement of plans, when given a choice of the second or third favourite, on purchase of candy bar	*64*	0.29	0.25	-0.53	2	1.00	4	2
Janiszewski & Chandon (2007)	4(2)	Compares measurement of plans to purchase the second-favourite candy bar vs. no measurement of plans, when given a choice of the top five favourites, on purchase of candy bar	*64*	0.46	0.25	-0.53	2	1.00	4	2
Lawrence & Ferguson (2012)	3(1)	Compares measurement of intention to quit smoking vs. no measurement of intention, on frequency of smoking	267	0.16	0.12	-2.62	5	2.33	4	2
Lawrence & Ferguson (2012)	3(2)	Compares measurement of intention to cut down on alcohol consumption vs. no measurement of intention, on number of alcohol units consumed	267	0.37	0.12	-2.62	5	2.67	2	2

Author(s) and publication year	Study (comparison)[a]	Comparison description	N[b]	*d*[c]	SE (adjusted)[d]	Mechanisms				
						Att. Acc.[e]	Att. Val.[f]	Cog. Diss.[g]	Ease of Rep.[h]	Corres.[i]
Lawrence & Ferguson (2012)	3(3)	Compares measurement of intention to engage in regular exercise vs. no measurement of intention, on frequency of exercise	267	-0.01	0.12	-0.95	4	2.67	4	2
Lawrence & Ferguson (2012)	3(4)	Compares measurement of intention to use a condom vs. no measurement of intention, on frequency of not using a condom	267	0.07	0.12	-0.95	4	3.33	4	2
Lawrence & Ferguson (2012)	3(5)	Compares measurement of intention to drive safely vs. no measurement of intention, on frequency of driving unsafely	267	0.31	0.12	-0.95	4	3.00	3	2
Levav & Fitzsimons (2006)	1	Compares measurement of likelihood of flossing teeth vs. measurement of likelihood of reading for pleasure, on frequency of flossing	97	0.42	0.20	0.59	5	2.00	3	3
Levav & Fitzsimons (2006)	2(1)	Compares measurement of likelihood of avoiding consumption of fatty foods vs. measurement of likelihood of consuming orange drinks, on choice of a high fat snack	51	1.59	0.55	-0.53	2	2.33	2	2
Levav & Fitzsimons (2006)	2(2)	Compares measurement of likelihood of consuming fatty foods vs. measurement of likelihood of consuming orange drinks, on choice of a high fat snack	48	-0.99	0.72	-0.53	2	3.00	4	2
Levav & Fitzsimons (2006)	2(3)	Compares measurement of likelihood of not consuming fatty foods vs. measurement of likelihood of consuming orange drinks, on choice of a high fat snack	50	0.92	0.56	-0.53	2	3.00	4	2
Levav & Fitzsimons (2006)	3(1)	Compares measurement of likelihood of reading for pleasure vs. measurement of likelihood of flossing teeth, on frequency of reading for pleasure	63	0.32	0.25	0.21	4	1.00	3	3
Levav & Fitzsimons (2006)	3(2)	Compares measurement of likelihood of flossing teeth vs. measurement of likelihood of reading for pleasure, on frequency of flossing	*31*	0.83	0.36	0.59	5	2.00	4	3
Manstead et al. (1983)	1(1)	Compares measurement of intention to breast feed or bottle feed baby in multiparous mothers vs. no measurement of intention in primiparous mothers, on incidence of breastfeeding	194	-0.62	0.22	0.59	5	4.00	3	3
Manstead et al. (1983)	1(2)	Compares measurement of intention to breast feed or bottle feed baby in primiparous mothers vs. no measurement of intention in primiparous mothers, on incidence of breastfeeding	191	-0.18	0.23	0.59	5	3.33	3	3
Morwitz & Fitzsimons (2004)	2(1)	Compares measurement of predicted likelihood of trying a Canadian candy bar vs. no measurement of predicted likelihood, on memory-based choice of target candy	72	-0.01	0.26	0.21	4	1.00	3	2
Morwitz & Fitzsimons (2004)	2(2)	Compares measurement of predicted likelihood of trying a Canadian candy bar vs. no measurement of predicted likelihood, on stimulus-based choice of target candy	95	-0.17	0.26	0.21	4	1.00	3	2
Morwitz & Fitzsimons (2004)	3(1)	Compares measurement of predicted likelihood of trying a Canadian candy bar vs. no measurement of predicted likelihood, on choice of negatively valenced target candy	56	-0.84	0.63	0.21	4	1.33	3	2

Author(s) and publication year	Study (comparison)[a]	Comparison description	N[b]	*d* [c]	SE (adjusted)[d]	Mechanisms				
						Att. Acc.[e]	Att. Val.[f]	Cog. Diss. [g]	Ease of Rep.[h]	Corres.[i]
Morwitz & Fitzsimons (2004)	3(2)	Compares measurement of predicted likelihood of trying a Canadian candy bar vs. no measurement of predicted likelihood, on choice of positively valenced target candy	39	1.17	0.40	0.21	4	1.33	3	2
Morwitz et al. (1993)	1(1)	Compares measurement of predicted timescale for purchasing next automobile vs. no measurement of predicted timescale, on automobile purchase incidence	8294	0.18	0.07	-0.16	3	2.00	3	3
Morwitz et al. (1993)	1(2)	Compares measurement of plans to acquire a personal computer vs. no measurement of plans, amongst participants with product experience, on PC ownership	4120	0.11	0.08	-0.16	3	2.00	3	1
Morwitz et al. (1993)	1(3)	Compares measurement of plans to acquire a personal computer vs. no measurement of plans, amongst participants with no product experience, on PC ownership	3711	0.21	0.15	-0.16	3	2.00	4	1
Obermiller & Spangenberg (2000)	1	Compares measurement of prediction to donate money to high school or college vs. no measurement of prediction (collapsed over no contact and unrelated prediction measurement conditions), on donation incidence	207	0.43	0.16	0.21	4	3.00	4	2
Obermiller et al. (1992)	1	Compares measurement of prediction to pledge support to business school vs. no measurement of prediction (no contact condition), on pledge incidence	157	-0.08	0.18	-0.16	3	3.00	3	3
Sandberg & Conner (2009)	1(1)	Compares measurement of prediction/intention to attend for a cervical smear, plus anticipated regret vs. no measurement of prediction/intention, on attendance for a cervical smear	2703	0.15	0.06	-5.10	5	4.00	3	3
Sandberg & Conner (2009)	1(2)	Compares measurement of prediction/intention to attend for a cervical smear vs. no measurement of prediction/intention, on attendance for a cervical smear	2748	0.17	0.06	-5.06	5	4.00	3	3
Sherman (1980)	1	Compares measurement of prediction to write a counterattitudinal essay vs. no measurement of prediction, on agreement to write a counterattitudinal essay	36	-0.75	0.39	-0.91	1	1.67	3	2
Sherman (1980)	3	Compares measurement of prediction to volunteer for the American Cancer Society vs. no measurement of prediction- behavior request only, on agreement to volunteer	91	1.26	0.43	0.59	5	3.00	4	3
Smith et al. (2003)	1(1)	Compares measurement of expectation to vote vs. no measurement of expectation, on voting turnout	588	0.00	0.09	0.21	4	2.33	3	4
Smith et al. (2003)	1(2)	Compares measurement of expectation to vote, plus reason for voting vs. no measurement of expectation, demographics questions only, on voting turnout	572	0.10	0.09	0.21	4	3.00	3	4
Spangenberg & Greenwald (1999)	Prelim.	Compares measurement of prediction to guess predominantly male, female or both names vs. no measurement of prediction, on relative number of female-name errors	193	0.70	0.23	-0.16	3	1.67	3	2
Spangenberg & Greenwald (1999)	1	Compares measurement of prediction to guess predominantly male, female or both names vs. no measurement of prediction, collapsed over gender monitoring condition, on relative number of female-name errors	77	0.15	0.11	-0.16	3	1.67	3	2

Author(s) and publication year	Study (comparison)[a]	Comparison description	N[b]	*d*[c]	SE (adjusted)[d]	Mechanisms				
						Att. Acc.[e]	Att. Val.[f]	Cog. Diss.[g]	Ease of Rep.[h]	Corres.[i]
Spangenberg & Greenwald (1999)	2(1)	Compares measurement of prediction to guess predominantly male, female or both names vs. no measurement of prediction, in participants with no prior experience on the task, on relative number of female-name errors	331	0.54	0.15	-0.16	3	1.67	3	2
Spangenberg & Greenwald (1999)	2(2)	Compares measurement of prediction to guess predominantly male, female or both names vs. no measurement of prediction, in participants with prior experience on the task, on relative number of female-name errors	200	0.36	0.15	-0.16	3	1.67	3	2
Spangenberg & Obermiller (1996)	1	Compares measurement of prediction to cheat on an exam or assignment vs. no measurement of prediction, on incidence of cheating on a take-home assignment	81	-0.53	0.26	-0.91	1	4.67	3	1
Spangenberg & Sprott (2006)	1(1)	Compares measurement of prediction to participate in a health and fitness assessment vs. measurement of prediction to tip at a restaurant after poor service, in high self-monitors, on signup for a health and fitness assessment	59	0.85	0.29	0.21	4	3.33	3	3
Spangenberg & Sprott (2006)	1(2)	Compares measurement of prediction to participate in a health and fitness assessment vs. measurement of prediction to tip at a restaurant after poor service, in low self-monitors, on signup for a health and fitness assessment	64	0.09	0.25	0.21	4	2.00	3	3
Spangenberg & Sprott (2006)	2(1)	Compares measurement of prediction to donate time to the American Cancer Society vs. measurement of prediction to tip at a restaurant after poor service, in high self-monitors, on signup for donation of time to assist the American Cancer Society	49	0.00	0.29	0.21	4	3.33	3	2
Spangenberg & Sprott (2006)	2(2)	Compares measurement of prediction to donate time to the American Cancer Society vs. measurement of prediction to tip at a restaurant after poor service, in low self-monitors, on signup for donation of time to assist the American Cancer Society	37	0.75	0.36	0.21	4	2.67	3	2
Spangenberg (1997)	1(1)	Compares measurement of expectation to use health club in the next week vs. no measurement of expectation, on number of health club visits up to 6 months after measurement	142	0.31	0.24	0.21	4	3.00	2	2
Spangenberg (1997)	1(2)	Compares measurement of expectation to use health club in the next week vs. no measurement of expectation, on incidence of visiting health club in 10 days after measurement	142	0.32	0.34	0.21	4	3.00	2	2
Spence et al. (2009)	1(1)	Compares measurement of intention to increase number of steps vs. no measurement of intention, in participants wearing a pedometer, on objectively measured number of steps	*31*	0.01	0.47	-0.16	3	2.67	3	3
Spence et al. (2009)	1(2)	Compares measurement of intention to increase number of steps vs. no measurement of intention, in participants wearing a pedometer, on self-reported walking	*31*	0.56	0.48	-0.16	3	2.67	3	3
Spence et al. (2009)	1(3)	Compares measurement of intention to increase number of steps vs. no measurement of intention, in participants not wearing a pedometer, on self-reported walking	*31*	0.46	0.35	-0.16	3	2.67	3	3
Sprott et al. (1999)	1	Compares measurement of prediction to recycle vs. measurement of prediction to tip at a restaurant after poor service plus other behaviors, on number of cans recycled	14	0.75	0.52	-2.03	5	3.00	3	2
Sprott et al. (1999)	2	Compares measurement of prediction to recycle vs. measurement of prediction to tip at a restaurant after poor service plus other behaviors, on number of cans recycled	126	-0.07	0.18	-2.03	5	3.00	3	2

Author(s) and publication year	Study (comparison)[a]	Comparison description	N[b]	*d* [c]	SE (adjusted)[d]	Mechanisms				
						Att. Acc.[e]	Att. Val.[f]	Cog. Diss. [g]	Ease of Rep.[h]	Corres.[i]
Sprott et al. (2003)	1(1)	Compares measurement of prediction to buy low or regular fat products vs. measurement of prediction regarding choice of newspaper, in participants with strong normative beliefs, on choice of a low-fat snack	39	0.94	0.47	0.21	4	3.67	3	1
Sprott et al. (2003)	1(2)	Compares measurement of prediction to buy low or regular fat products vs. measurement of prediction regarding choice of newspaper, in participants with weak normative beliefs, on choice of a low-fat snack	41	0.10	0.37	0.21	4	2.67	3	1
Sprott et al. (2003)	2(1)	Compares measurement of prediction to participate in a health and fitness assessment vs. measurement of prediction to tip at a restaurant after poor service, in participants with strong normative beliefs, on signup for a health and fitness assessment	65	0.70	0.30	0.21	4	3.67	3	2
Sprott et al. (2003)	2(2)	Compares measurement of prediction to participate in a health and fitness assessment vs. measurement of prediction to tip at a restaurant after poor service, in participants with weak normative beliefs, on signup for a health and fitness assessment	72	0.29	0.30	0.21	4	1.33	3	2
Sprott et al. (2004)	1	Compares measurement of prediction to participate in a health and fitness assessment vs. measurement of prediction to tip at a restaurant after poor service, on signup for a health and fitness assessment	243	0.62	0.15	-0.16	3	2.67	3	2
Sprott et al. (2004)	2(1)	Compares measurement of general prediction to participate in health and fitness activities vs. measurement of prediction to tip at a restaurant after poor service, on signup for a health and fitness assessment	120	0.26	0.27	-0.16	3	2.33	3	1
Sprott et al. (2004)	2(2)	Compares measurement of specific prediction to participate in a health and fitness assessment vs. measurement of prediction to tip at a restaurant after poor service, on signup for a health and fitness assessment	121	0.66	0.27	-0.16	3	2.67	3	2
Van Kerckhove, Geuens & Vermeir (2012)	1(1)	Compares measurement of likelihood of trying the presented candy bars if they became available vs. measurement of attitudes towards the presented candy bars becoming available, in participants also given an attitude accessibility task, on choice of candy	92	0.49	0.25	-0.53	2	1.33	3	2
Van Kerckhove, Geuens & Vermeir (2012)	1(2)	Compares measurement of likelihood of trying the presented candy bars if they became available vs. measurement of attitudes towards the presented candy bars becoming available, in participants not given an attitude accessibility task, on choice of candy	87	0.55	0.28	-0.53	2	1.33	3	2
Van Kerckhove, Geuens & Vermeir (2012)	2A	Compares measurement of likelihood of trying the presented candy bars if they became available vs. no measurement of likelihood, on initial choice of candy	87	0.87	0.27	-0.53	2	1.33	3	2
Van Kerckhove, Geuens & Vermeir (2012)	2B	Compares measurement of likelihood of purchasing the presented candy brands vs. measurement of attitudes towards the presented candy brands, on initial choice of candy	106	-0.09	0.31	-0.53	2	1.33	3	2
Van Kerckhove, Geuens & Vermeir (2012)	3(1)	Compares measurement of likelihood of trying the presented candy bars if they became available vs. measurement of attitudes towards the presented candy bars becoming available, on initial choice of candy after a 10 minute filler task	115	1.16	0.25	-0.53	2	1.00	3	2
Van Kerckhove, Geuens & Vermeir (2012)	3(2)	Compares measurement of likelihood of trying the presented candy bars if they became available vs. measurement of attitudes towards the presented candy bars becoming available, on initial choice of candy after no filler task	123	0.37	0.23	-0.53	2	1.33	3	2
Williams et al. (2004)	1(1)	Compares measurement of likelihood of eating fatty foods vs. measurement of likelihood of flossing, on frequency of eating fatty foods	*137*	-0.80	0.25	-0.53	2	2.00	4	3

Author(s) and publication year	Study (comparison)[a]	Comparison description	N[b]	*d* [c]	SE (adjusted)[d]	Mechanisms				
						Att. Acc.[e]	Att. Val.[f]	Cog. Diss. [g]	Ease of Rep.[h]	Corres.[i]
Williams et al. (2004)	1(2)	Compares measurement of likelihood of eating fatty foods, vs. measurement of likelihood of flossing, ostensibly sponsored by an objective source, on frequency of eating fatty foods	*137*	-0.44	0.24	-0.53	2	3.00	4	3
Williams et al. (2004)	1(3)	Compares measurement of likelihood of eating fatty foods vs. measurement of likelihood of flossing, ostensibly sponsored by a self-interested source, on frequency of eating fatty foods	*137*	-0.20	0.24	-0.53	2	1.33	4	3
Williams et al. (2004)	1(4)	Compares measurement of likelihood of flossing vs. measurement of likelihood of eating fatty foods, on frequency of flossing	*137*	0.96	0.26	0.21	4	2.00	3	3
Williams et al. (2004)	1(5)	Compares measurement of likelihood of flossing, vs. measurement of likelihood of eating fatty foods, ostensibly sponsored by an objective source, on frequency of flossing	*137*	0.41	0.24	0.21	4	3.00	3	3
Williams et al. (2004)	1(6)	Compares measurement of likelihood of flossing vs. measurement of likelihood of eating fatty foods, ostensibly sponsored by a self-interested source, on frequency of flossing	*137*	-1.25	0.27	0.21	4	1.33	3	3
Williams et al. (2004)	2(1)	Compares measurement of likelihood of eating fatty foods vs. measurement of likelihood of flossing, under normal cognitive capacity, on frequency of eating fatty foods	*58*	-0.89	0.39	-0.53	2	2.00	4	3
Williams et al. (2004)	2(2)	Compares measurement of likelihood of eating fatty foods, vs. measurement of likelihood of flossing, ostensibly sponsored by an objective source, under normal cognitive capacity, on frequency of eating fatty foods	*58*	-0.89	0.39	-0.53	2	3.00	4	3
Williams et al. (2004)	2(3)	Compares measurement of likelihood of eating fatty foods vs. measurement of likelihood of flossing, ostensibly sponsored by a self-interested source, under normal cognitive capacity, on frequency of eating fatty foods	*58*	0.89	0.39	-0.53	2	1.33	4	3
Williams et al. (2004)	2(4)	Compares measurement of likelihood of flossing vs. measurement of likelihood of eating fatty foods, under normal cognitive capacity, on frequency of flossing	*58*	1.53	0.43	0.21	4	2.00	3	3
Williams et al. (2004)	2(5)	Compares measurement of likelihood of flossing, vs. measurement of likelihood of eating fatty foods, ostensibly sponsored by an objective source, under normal cognitive capacity, on frequency of flossing	*58*	1.38	0.42	0.21	4	3.00	3	3
Williams et al. (2004)	2(6)	Compares measurement of likelihood of flossing vs. measurement of likelihood of eating fatty foods, ostensibly sponsored by a self-interested source, under normal cognitive capacity, on frequency of flossing	*58*	-0.36	0.37	0.21	4	1.33	3	3
Williams et al. (2004)	2(7)	Compares measurement of likelihood of flossing vs. measurement of likelihood of eating fatty foods, ostensibly sponsored by a self-interested source, under constrained cognitive capacity, on frequency of flossing	*58*	1.25	0.32	0.21	4	1.00	3	3
Williams et al. (2004)	3(1)	Compares measurement of likelihood of eating fatty foods vs. measurement of likelihood of watching TV, under normal cognitive capacity, on frequency of eating fatty foods	*65*	-1.14	0.40	-0.53	2	2.33	3	2
Williams et al. (2004)	3(2)	Compares measurement of likelihood of eating fatty foods vs. measurement of likelihood of watching TV, ostensibly sponsored by a self-interested source, under normal cognitive capacity, on frequency of eating fatty foods	*65*	-0.15	0.42	-0.53	2	1.33	3	2

Author(s) and publication year	Study (comparison)[a]	Comparison description	N[b]	*d* [c]	SE (adjusted)[d]	Mechanisms				
						Att. Acc.[e]	Att. Val.[f]	Cog. Diss. [g]	Ease of Rep.[h]	Corres.[i]
Williams et al. (2004)	3(3)	Compares measurement of likelihood of eating fatty foods vs. measurement of likelihood of watching TV, under constrained cognitive capacity, on frequency of eating fatty foods	*65*	-0.70	0.36	-0.53	2	1.33	3	2
Williams et al. (2004)	3(4)	Compares measurement of likelihood of eating fatty foods vs. measurement of likelihood of watching TV, ostensibly sponsored by a self-interested source, under constrained cognitive capacity, on frequency of eating fatty foods	*65*	-0.80	0.36	-0.53	2	1.00	3	2
Williams et al. (2004)	4(1)	Compares measurement of likelihood of participating in the Teach for America program vs. no measurement of likelihood, plus exposure to abstract on the mere-measurement effect, on signup for additional information	73	0.09	0.48	0.59	5	1.00	3	1
Williams et al. (2004)	4(2)	Compares measurement of likelihood of participating in the Teach for America program vs. no measurement of likelihood, plus exposure to abstract on attitude stability, on signup for additional information	70	0.76	0.45	0.59	5	1.67	3	1
Williams et al. (2006)	1(1)	Compares measurement of likelihood of using illegal drugs vs. measurement of likelihood of exercising, on frequency of illegal drug use	167	0.31	0.16	-0.91	1	2.67	3	3
Williams et al. (2006)	1(2)	Compares measurement of likelihood of exercising vs. measurement of likelihood of using illegal drugs, on frequency of exercise	167	0.25	0.15	0.21	4	4.00	4	3

Note. [a]The number in parentheses indicates where multiple effect sizes were included for a single study, when studies reported more than one behavioral observation for the same group of participants that were equally integral to the moderator analyses. The description of the comparison is included in the comparison description column. [b]Ns in italics were estimated from total participant sample size. [c]*d* = Standardized mean difference effect size with Hedge's adjustment. [d]When multiple effect sizes for non-independent comparisons were included, the sample sizes used to calculate the standard errors for each group were divided by the number of times they were included, to avoid underestimating the error variance associated with each effect size. [e]Accessibility of attitudes towards behavior in the participant sample, such that higher scores indicate greater accessibility of more positive attitudes. [f]Valence of attitude towards behavior in the participant sample, such that higher scores indicate more positive attitudes. [g]Average of items measuring the likely degree of discomfort experienced by participants at the time of prediction, if their past behavior was not consistent with their predictions or intentions about their future behaviour/ the likely degree of discomfort experienced by participants at the time of prediction, if their future behavior was not consistent with their predictions or intentions regarding their future behaviour/ and the likely degree of discomfort experienced by participants at the time of opportunity for future behaviour, if their future behavior was not consistent with their predictions or intentions regarding their future behaviour. [h]Congruence of match between likely attitude valence and question frame, such that higher scores indicate greater congruence/ease of representation. [i]Match between the measure of intention/prediction and behavior on action, target, context and time, such that higher scores indicate greater correspondence.
