# Supplementary material for: The Impact of Asking Intention or Self-Prediction Questions on Subsequent Behavior: A Meta-Analysis
Source: Pers Soc Psychol Rev. 2015 Jul 10;20(3):245–68. doi: 10.1177/1088868315592334 (PMC4931712; doi:10.1177/1088868315592334)
Supplement: Supplementary material [file 10.1177_1088868315592334_AppendixB.pdf]

Appendix B

Moderator Coding of Question, Behavior and Methodological Characteristics for Studies Included in the Meta-Analysis.

Author(s) and publication year	Study (comparison)[a]	Question characteristics					Behaviour characteristics						Methodological characteristics					
		Q Type[b]	TPB[c]	AR[d]	N Pred/Int Qs[e]	N Beh. Qs[f]	Beh. Exp.[g]	Beh. Dom.[h]	Social Des.[i]	Diff. of Beh.[j]	Beh. Freq.[k]	Beh. Meas.[l]	Time Int.[m]	Pers. Contact[n]	Res. Setting[o]	RR[p]	Incent.[q]	Sample Type[r]
Ayres et al. (2013)	1	MIX	TPB	AR	2	8	NO	HEA	4	2	OT	OB	0	IMP	FD	1.00	Y	NS
Chandon et al. (2004)	1(1)	MIX	NO	NO	2	2	HI	CON	2	2	REP	OB	270	PER	FD	1.00	N	NS
Chandon et al. (2004)	1(2)	MIX	NO	NO	2	2	HI	CON	2	2	REP	OB	60	PER	FD	1.00	N	NS
Chandon et al. (2011)	1(1)	PRED	NO	NO	1	4	VAR	HEA	4	3	REP	SR	7	IMP	FD	1.00	N	S
Chandon et al. (2011)	1(2)	PRED	NO	NO	1	4	VAR	OTH	3	1	REP	SR	7	IMP	FD	1.00	N	S
Chapman (2001)	2(1)	PRED	NO	NO	6	6	VAR	PRO	3	2	OT	OB	1	IMP	FD	1.00	N	S
Chapman (2001)	2(2)	PRED	NO	NO	6	6	VAR	PRO	3	2	OT	OB	6	IMP	FD	1.00	N	S
Chapman (2001)	2(3)	PRED	NO	NO	1	1	VAR	PRO	3	2	OT	OB	1	IMP	FD	1.00	N	S
Chapman (2001)	2(4)	PRED	NO	NO	1	1	VAR	PRO	3	2	OT	OB	6	IMP	FD	1.00	N	S
Cioffi & Garner (1998)	1(1)	INT	NO	NO	1	1	VAR	PRO	4	4	OT	OB	7	IMP	FD	0.30	N	S
Cioffi & Garner (1998)	1(2)	INT	NO	NO	1	1	VAR	PRO	4	4	OT	OB	7	IMP	FD	0.19	N	S
Cioffi & Garner (1998)	1(3)	INT	NO	NO	1	1	VAR	PRO	4	4	OT	OB	7	IMP	FD	0.64	N	S
Conner et al. (2011)	1	MIX	TPB	NO	3	22	VAR	HEA	5	3	OT	OB	120	IMP	FD	0.47	N	NS
Conner et al. (2011)	2	MIX	TPB	AR	3	34	VAR	HEA	5	2	OT	OB	60	IMP	FD	0.72	N	NS
Cox et al. (2012)	1(1)	PRED	NO	NO	3	19	NO	HEA	5	5	OT	OB	0	IMP	FD	1.00	Y	NS
Cox et al. (2012)	1(2)	PRED	NO	NO	3	19	NO	HEA	5	2	OT	OB	0	IMP	FD	1.00	Y	NS
Fitzsimons & Williams (2000)	1(1)	PRED	NO	NO	1	7	NO	CON	3	1	OT	OB	0	IMP	LB	1.00	Y	MIX
Fitzsimons & Williams (2000)	1(2)	PRED	NO	NO	1	7	NO	CON	3	1	OT	OB	0	IMP	LB	1.00	Y	MIX
Fitzsimons & Williams (2000)	1(3)	PRED	NO	NO	1	7	NO	CON	3	1	OT	OB	0	IMP	LB	1.00	Y	MIX
Fitzsimons & Williams (2000)	1(4)	PRED	NO	NO	1	7	NO	CON	3	1	OT	OB	0	IMP	LB	1.00	Y	MIX
Fitzsimons & Williams (2000)	2(1)	PRED	NO	NO	1	6	VAR	PRO	5	2	OT	OB	0	IMP	LB	1.00	Y	S
Fitzsimons & Williams (2000)	2(2)	PRED	NO	NO	1	6	VAR	PRO	5	2	OT	OB	0	IMP	LB	1.00	Y	S
Fitzsimons & Williams (2000)	2(3)	PRED	NO	NO	1	6	VAR	PRO	5	2	OT	OB	0	IMP	LB	1.00	Y	S
Fitzsimons & Williams (2000)	2(4)	PRED	NO	NO	1	6	VAR	PRO	5	2	OT	OB	0	IMP	LB	1.00	Y	S
Fitzsimons et al. (2007)	1	PRED	NO	NO	1	1	VAR	UND	1	2	REP	OB	112	IMP	FD	1.00	Y	S
Fitzsimons et al. (2007)	4(1)	PRED	NO	NO	1	1	VAR	UND	3	1	REP	SR	7	IMP	FD	1.00	Y	S
Fitzsimons et al. (2007)	4(2)	PRED	NO	NO	1	1	VAR	UND	2	1	REP	SR	7	IMP	FD	1.00	Y	S
Godin et al. (2008)	1(1)	MIX	TPB	AR	3	77	HI	PRO	4	4	REP	OB	365	IMP	FD	0.82	N	NS
Godin et al. (2008)	1(2)	MIX	TPB	AR	3	77	HI	PRO	4	4	REP	OB	180	IMP	FD	0.82	N	NS
Godin et al. (2010)	1(1)	MIX	NO	AR	3	6	NO	PRO	4	5	REP	OB	365	IMP	FD	0.40	N	NS
Godin et al. (2010)	1(2)	MIX	NO	AR	3	6	NO	PRO	4	5	REP	OB	180	IMP	FD	0.40	N	NS
Godin et al. (2010)	1(3)	MIX	NO	AR	3	3	NO	PRO	4	5	REP	OB	365	IMP	FD	0.42	N	NS
Godin et al. (2010)	1(4)	MIX	NO	AR	3	3	NO	PRO	4	5	REP	OB	180	IMP	FD	0.42	N	NS
Godin et al. (2011)	1	MIX	TPB	AR	3	47	VAR	HEA	5	5	REP	SR	90	IMP	FD	1.00	N	MIX
Greenwald et al. (1987)	1	PRED	NO	NO	1	4	NO	PRO	5	2	OT	MIX	2	PER	FD	1.00	N	S
Greenwald et al. (1987)	2	PRED	NO	NO	1	4	VAR	PRO	5	3	OT	MIX	1	PER	FD	1.00	N	S

Author(s) and publication year	Study (comparison)[a]	Question characteristics					Behaviour characteristics						Methodological characteristics					
		Q Type[b]	TPB[c]	AR[d]	N Pred/Int Qs[e]	N Beh. Qs[f]	Beh. Exp.[g]	Beh. Dom.[h]	Social Des.[i]	Diff. of Beh.[j]	Beh. Freq.[k]	Beh. Meas.[l]	Time Int.[m]	Pers. Contact[n]	Res. Setting[o]	RR[p]	Incent.[q]	Sample Type[r]
Janiszewski & Chandon (2007)	4(1)	MIX	NO	NO	1	17	VAR	CON	3	1	OT	OB	0	IMP	LB	1.00	Y	S
Janiszewski & Chandon (2007)	4(2)	MIX	NO	NO	1	17	VAR	CON	3	1	OT	OB	0	IMP	LB	1.00	Y	S
Lawrence & Ferguson (2012)	3(1)	INT	NO	NO	1	2	VAR	HEA	5	5	REP	SR	60	IMP	FD	0.51	N	S
Lawrence & Ferguson (2012)	3(2)	INT	NO	NO	1	2	VAR	HEA	4	4	REP	SR	60	IMP	FD	0.51	N	S
Lawrence & Ferguson (2012)	3(3)	INT	NO	NO	1	2	VAR	HEA	4	3	REP	SR	60	IMP	FD	0.51	N	S
Lawrence & Ferguson (2012)	3(4)	INT	NO	NO	1	2	VAR	HEA	4	3	REP	SR	60	IMP	FD	0.51	N	S
Lawrence & Ferguson (2012)	3(5)	INT	NO	NO	1	2	VAR	OTH	5	2	REP	SR	60	IMP	FD	0.51	N	S
Levav & Fitzsimons (2006)	1	PRED	NO	NO	1	1	VAR	HEA	4	2	REP	SR	14	IMP	FD	1.00	N	S
Levav & Fitzsimons (2006)	2(1)	PRED	NO	NO	1	1	VAR	UND	3	3	OT	SR	0	IMP	LB	1.00	N	S
Levav & Fitzsimons (2006)	2(2)	PRED	NO	NO	1	1	VAR	UND	3	3	OT	SR	0	IMP	LB	1.00	N	S
Levav & Fitzsimons (2006)	2(3)	PRED	NO	NO	1	1	VAR	UND	3	3	OT	SR	0	IMP	LB	1.00	N	S
Levav & Fitzsimons (2006)	3(1)	PRED	NO	NO	1	1	VAR	OTH	4	1	REP	SR	7	IMP	FD	1.00	N	S
Levav & Fitzsimons (2006)	3(2)	PRED	NO	NO	1	1	VAR	HEA	4	2	REP	SR	7	IMP	FD	1.00	N	S
Manstead et al. (1983)	1(1)	INT	TPB	NO	1	37	VAR	PRO	5	3	OT	SR	180	IMP	FD	1.00	N	NS
Manstead et al. (1983)	1(2)	INT	TPB	NO	1	40	NO	PRO	5	4	OT	SR	180	IMP	FD	1.00	N	NS
Morwitz & Fitzsimons (2004)	2(1)	PRED	NO	NO	1	3	NO	CON	3	1	OT	OB	0	IMP	LB	1.00	N	S
Morwitz & Fitzsimons (2004)	2(2)	PRED	NO	NO	1	3	NO	CON	3	1	OT	OB	0	IMP	LB	1.00	N	S
Morwitz & Fitzsimons (2004)	3(1)	PRED	NO	NO	1	7	NO	CON	3	1	OT	OB	0	IMP	LB	1.00	N	S
Morwitz & Fitzsimons (2004)	3(2)	PRED	NO	NO	1	7	NO	CON	3	1	OT	OB	0	IMP	LB	1.00	N	S
Morwitz et al. (1993)	1(1)	PRED	NO	NO	1	2	VAR	CON	3	4	OT	SR	180	IMP	FD	1.00	N	NS
Morwitz et al. (1993)	1(2)	INT	NO	NO	1	2	HI	CON	3	3	OT	SR	180	IMP	FD	1.00	N	NS
Morwitz et al. (1993)	1(3)	INT	NO	NO	1	2	NO	CON	3	4	OT	SR	180	IMP	FD	1.00	N	NS
Obermiller & Spangenberg (2000)	1	PRED	NO	NO	1	1	VAR	PRO	5	2	OT	OB	85	PER	FD	1.00	N	NS
Obermiller et al. (1992)	1	PRED	NO	NO	1	2	VAR	PRO	5	2	OT	OB	28	PER	FD	1.00	N	NS
Sandberg & Conner (2009)	1(1)	MIX	TPB	AR	2	19	VAR	HEA	5	5	OT	OB	120	IMP	FD	0.13	Y	NS
Sandberg & Conner (2009)	1(2)	MIX	TPB	NO	2	13	VAR	HEA	5	5	OT	OB	120	IMP	FD	0.14	Y	NS
Sherman (1980)	1	PRED	NO	NO	1	1	NO	OTH	4	2	OT	OB	14	PER	FD	1.00	N	S
Sherman (1980)	3	PRED	NO	NO	1	1	VAR	PRO	5	3	OT	OB	3	PER	FD	1.00	N	NS
Smith et al. (2003)	1(1)	PRED	NO	NO	1	3	HI	PRO	5	2	OT	OB	2	PER	FD	1.00	N	NS
Smith et al. (2003)	1(2)	PRED	NO	NO	1	4	HI	PRO	5	2	OT	OB	2	PER	FD	1.00	N	NS
Spangenberg & Greenwald (1999)	Prelim.	PRED	NO	NO	1	1	NO	OTH	3	1	REP	OB	0	IMP	LB	1.00	Y	S
Spangenberg & Greenwald (1999)	1	PRED	NO	NO	1	1	NO	OTH	3	1	REP	OB	0	IMP	LB	1.00	Y	S
Spangenberg & Greenwald (1999)	2(1)	PRED	NO	NO	1	1	NO	OTH	3	1	REP	OB	0	IMP	LB	1.00	Y	S
Spangenberg & Greenwald (1999)	2(2)	PRED	NO	NO	1	1	NO	OTH	3	1	REP	OB	0	IMP	LB	1.00	Y	S
Spangenberg & Obermiller (1996)	1	PRED	NO	NO	1	1	VAR	UND	1	3	OT	OB	2	IMP	FD	1.00	Y	S
Spangenberg & Sprott (2006)	1(1)	PRED	NO	NO	1	1	VAR	HEA	5	2	OT	OB	0	IMP	LB	1.00	Y	S
Spangenberg & Sprott (2006)	1(2)	PRED	NO	NO	1	1	VAR	HEA	5	2	OT	OB	0	IMP	LB	1.00	Y	S
Spangenberg & Sprott (2006)	2(1)	PRED	NO	NO	1	1	VAR	PRO	5	3	OT	OB	0	IMP	LB	1.00	Y	S
Spangenberg & Sprott (2006)	2(2)	PRED	NO	NO	1	1	VAR	PRO	5	3	OT	OB	0	IMP	LB	1.00	Y	S
Spangenberg (1997)	1(1)	PRED	NO	NO	1	2	HI	HEA	5	4	REP	OB	180	PER	FD	1.00	N	NS
Spangenberg (1997)	1(2)	PRED	NO	NO	1	2	HI	HEA	5	4	REP	OB	10	PER	FD	1.00	N	NS
Spence et al. (2009)	1(1)	INT	NO	NO	1	17	NO	HEA	5	1	REP	OB	7	IMP	FD	1.00	N	S
Spence et al. (2009)	1(2)	INT	NO	NO	1	17	NO	HEA	5	1	REP	SR	7	IMP	FD	1.00	N	S
Spence et al. (2009)	1(3)	INT	NO	NO	1	17	NO	HEA	5	1	REP	SR	7	IMP	FD	1.00	N	S

Author(s) and publication year	Study (comparison)[a]	Question characteristics					Behaviour characteristics						Methodological characteristics					
		Q Type[b]	TPB[c]	AR[d]	N Pred/Int Qs[e]	N Beh. Qs[f]	Beh. Exp.[g]	Beh. Dom.[h]	Social Des.[i]	Diff. of Beh.[j]	Beh. Freq.[k]	Beh. Meas.[l]	Time Int.[m]	Pers. Contact[n]	Res. Setting[o]	RR[p]	Incent.[q]	Sample Type[r]
Sprott et al. (1999)	1	PRED	NO	NO	1	1	VAR	PRO	5	2	REP	OB	77	IMP	FD	0.60	N	S
Sprott et al. (1999)	2	PRED	NO	NO	1	2	VAR	PRO	5	2	REP	OB	105	IMP	FD	0.60	N	S
Sprott et al. (2003)	1(1)	PRED	TPB	NO	1	1	VAR	HEA	3	3	OT	OB	0	IMP	LB	1.00	N	S
Sprott et al. (2003)	1(2)	PRED	TPB	NO	1	1	VAR	HEA	3	3	OT	OB	0	IMP	LB	1.00	N	S
Sprott et al. (2003)	2(1)	PRED	TPB	NO	1	6	VAR	HEA	5	3	OT	OB	0	IMP	LB	1.00	N	S
Sprott et al. (2003)	2(2)	PRED	TPB	NO	1	6	VAR	HEA	4	3	OT	OB	0	IMP	LB	1.00	N	S
Sprott et al. (2004)	1	PRED	NO	NO	1	1	VAR	HEA	5	2	OT	OB	0	IMP	LB	1.00	N	S
Sprott et al. (2004)	2(1)	PRED	NO	NO	1	1	VAR	HEA	5	2	OT	OB	0	IMP	LB	1.00	N	S
Sprott et al. (2004)	2(2)	PRED	NO	NO	1	1	VAR	HEA	5	2	OT	OB	0	IMP	LB	1.00	N	S
Van Kerckhove, Geuens & Vermeir (2012)	1(1)	PRED	NO	NO	1	2	NO	CON	3	1	OT	OB	0	IMP	LB	1.00	N	S
Van Kerckhove, Geuens & Vermeir (2012)	1(2)	PRED	NO	NO	1	2	NO	CON	3	1	OT	OB	0	IMP	LB	1.00	N	S
Van Kerckhove, Geuens & Vermeir (2012)	2A	PRED	NO	NO	1	2	NO	CON	3	1	OT	OB	0	IMP	LB	1.00	N	S
Van Kerckhove, Geuens & Vermeir (2012)	2B	PRED	NO	NO	1	4	VAR	CON	3	1	OT	OB	0	IMP	LB	1.00	N	S
Van Kerckhove, Geuens & Vermeir (2012)	3(1)	PRED	NO	NO	1	2	NO	CON	3	1	OT	OB	0	IMP	LB	1.00	N	MIX
Van Kerckhove, Geuens & Vermeir (2012)	3(2)	PRED	NO	NO	1	2	NO	CON	3	1	OT	OB	0	IMP	LB	1.00	N	MIX
Williams et al. (2004)	1(1)	PRED	NO	NO	1	1	VAR	UND	1	2	REP	SR	7	IMP	FD	1.00	N	S
Williams et al. (2004)	1(2)	PRED	NO	NO	1	1	VAR	UND	1	2	REP	SR	7	IMP	FD	1.00	N	S
Williams et al. (2004)	1(3)	PRED	NO	NO	1	1	VAR	UND	1	2	REP	SR	7	IMP	FD	1.00	N	S
Williams et al. (2004)	1(4)	PRED	NO	NO	1	1	VAR	HEA	4	2	REP	SR	7	IMP	FD	1.00	N	S
Williams et al. (2004)	1(5)	PRED	NO	NO	1	1	VAR	HEA	4	2	REP	SR	7	IMP	FD	1.00	N	S
Williams et al. (2004)	1(6)	PRED	NO	NO	1	1	VAR	HEA	4	2	REP	SR	7	IMP	FD	1.00	N	S
Williams et al. (2004)	2(1)	PRED	NO	NO	1	1	VAR	UND	1	2	REP	SR	7	IMP	FD	1.00	N	S
Williams et al. (2004)	2(2)	PRED	NO	NO	1	1	VAR	UND	1	2	REP	SR	7	IMP	FD	1.00	N	S
Williams et al. (2004)	2(3)	PRED	NO	NO	1	1	VAR	UND	1	2	REP	SR	7	IMP	FD	1.00	N	S
Williams et al. (2004)	2(4)	PRED	NO	NO	1	1	VAR	HEA	4	2	REP	SR	7	IMP	FD	1.00	N	S
Williams et al. (2004)	2(5)	PRED	NO	NO	1	1	VAR	HEA	4	2	REP	SR	7	IMP	FD	1.00	N	S
Williams et al. (2004)	2(6)	PRED	NO	NO	1	1	VAR	HEA	4	2	REP	SR	7	IMP	FD	1.00	N	S
Williams et al. (2004)	2(7)	PRED	NO	NO	1	1	VAR	HEA	4	2	REP	SR	7	IMP	FD	1.00	N	S
Williams et al. (2004)	3(1)	PRED	NO	NO	1	1	VAR	UND	1	2	OT	OB	7	IMP	LB	1.00	N	MIX
Williams et al. (2004)	3(2)	PRED	NO	NO	1	1	VAR	UND	1	2	OT	OB	7	IMP	LB	1.00	N	MIX
Williams et al. (2004)	3(3)	PRED	NO	NO	1	1	VAR	UND	1	2	OT	OB	7	IMP	LB	1.00	N	MIX
Williams et al. (2004)	3(4)	PRED	NO	NO	1	1	VAR	UND	1	2	OT	OB	7	IMP	LB	1.00	N	MIX
Williams et al. (2004)	4(1)	PRED	NO	NO	1	4	VAR	PRO	4	1	OT	OB	0	IMP	LB	1.00	N	MIX
Williams et al. (2004)	4(2)	PRED	NO	NO	1	4	VAR	PRO	4	1	OT	OB	0	IMP	LB	1.00	N	MIX
Williams et al. (2006)	1(1)	PRED	NO	NO	1	1	VAR	UND	1	4	REP	SR	60	IMP	FD	1.00	Y	S
Williams et al. (2006)	1(2)	PRED	NO	NO	1	1	VAR	HEA	3	3	REP	SR	60	IMP	FD	1.00	Y	S

Note. [a]The number in parentheses indicates where multiple effect sizes were included for a single study, when studies reported more than one behavioral observation for the same group of participants that were equally integral to the moderator analyses. See Appendix A for descriptions of the comparisons in this column. [b]The type of question used to invoke the QBE, where MIX = Mixed, PRED = Prediction/Expectation, INT = Intention. [c]Whether additional questions based on the Theory of Planned Behavior were included, where TPB = Additional Theory of Planned

Behavior items present, NO = No additional Theory of Planned Behavior items present. [d]Whether additional questions measuring anticipated regret were included, where AR = Anticipated Regret items present, NO = No Anticipated Regret items present. [e]The number of items asking participants to report behavioral intentions or predictions. [f]The total number of items relating to the behavior. [g]The participant sample's likely level of experience with the behavior, where HI = High Experience, NO = No Experience, VAR = Varied Experience. [h]The type of behavior targeted, where HEA = Health behaviors, PRO = Prosocial behaviors, UND = Undesirable or risky behaviors, CON = Consumer behaviors, and OTH = Unclassifed other behaviors. [i]Social desirability of behavior, such that higher scores indicate greater desirability. [j]Difficulty to perform the behavior, such that higher scores indicate greater difficulty. [k]Whether the behavior was measured as a one-time occurrence or repeated behavior, where OT = One-time and REP = Repeated. [l]Whether behavior was measured using objective or self-report measures, where OB = Objective measures, and SR = Self-report measures. [m]Time between measurement of intention/prediction and measurement of behavior in days. [n]Whether intentions/predictions were reported via personal contact with the experimenter, where PER = Personal Contact and IMP = Impersonal Contact. [o]The setting in which behavior was measured, where LB = Behavior measured in the laboratory, and FD = Behavior measured in the field. [p]Response rate to the instrument (e.g. questionnaire) measuring intentions/predictions. Scores of 1 indicate lab-based studies with 100% response rates. [q]Whether an incentive was given for participation, where Y = Yes, and N = No. [r]The nature of the participant sample, where S = Student, NS = Non-student, and MIX= Mixed or unreported sample type.
